# Modularity of the hydrophobic core and evolution of functional diversity in fold A glycosyltransferases

**DOI:** 10.1016/j.jbc.2022.102212

**Published:** 2022-06-30

**Authors:** Aarya Venkat, Daniel Tehrani, Rahil Taujale, Wayland Yeung, Nathan Gravel, Kelley W. Moremen, Natarajan Kannan

**Affiliations:** 1 Department of Biochemistry and Molecular Biology, University of Georgia, Athens, Georgia, USA; 2 Complex Carbohydrate Research Center (CCRC), University of Georgia, Athens, Georgia, USA; 3 Institute of Bioinformatics, University of Georgia, Athens, Georgia, USA

**Keywords:** glycobiology, glycosyltransferase, protein evolution, structure–function, bioinformatics, GT, glycosyltransferase, GT-A, fold-A glycosyltransferase, HMM, hidden Markov model, MD, molecular dynamics, PBC, phosphate-binding cassette, PDB, Protein Data Bank

## Abstract

Hydrophobic cores are fundamental structural properties of proteins typically associated with protein folding and stability; however, how the hydrophobic core shapes protein evolution and function is poorly understood. Here, we investigated the role of conserved hydrophobic cores in fold-A glycosyltransferases (GT-As), a large superfamily of enzymes that catalyze formation of glycosidic linkages between diverse donor and acceptor substrates through distinct catalytic mechanisms (inverting *versus* retaining). Using hidden Markov models and protein structural alignments, we identify similarities in the phosphate-binding cassette (PBC) of GT-As and unrelated nucleotide-binding proteins, such as UDP-sugar pyrophosphorylases. We demonstrate that GT-As have diverged from other nucleotide-binding proteins through structural elaboration of the PBC and its unique hydrophobic tethering to the F-helix, which harbors the catalytic base (xED-Asp). While the hydrophobic tethering is conserved across diverse GT-A fold enzymes, some families, such as B3GNT2, display variations in tethering interactions and core packing. We evaluated the structural and functional impact of these core variations through experimental mutational analysis and molecular dynamics simulations and find that some of the core mutations (T336I in B3GNT2) increase catalytic efficiency by modulating the conformational occupancy of the catalytic base between “D-in” and acceptor-accessible “D-out” conformation. Taken together, our studies support a model of evolution in which the GT-A core evolved progressively through elaboration upon an ancient PBC found in diverse nucleotide-binding proteins, and malleability of this core provided the structural framework for evolving new catalytic and substrate-binding functions in extant GT-A fold enzymes.

Glycosyltransferases (GTs) are a diverse family of enzymes that catalyze the formation of glycosidic linkages between sugars and other macromolecules (1). These enzymes are found across the tree of life and are involved in a number of critical cellular functions through post-translational modifications, including protein folding, signaling, and stability (1). Misregulation, or aberrant glycosylation, is implicated in a wide range of diseases, including Alzheimer’s, Parkinson’s, muscular dystrophies, and human cancers (2, 3, 4, 5, 6, 7). Based on the catalytic mechanism, GTs are broadly classified as “inverting” or “retaining” based on the stereochemistry of the glycosidic bond they generate (Fig. S1). Inverting GTs generally employ a direct S_N_2 displacement mechanism with a protein-associated catalytic base that deprotonates the acceptor nucleophile hydroxyl leading to attack on the anomeric center and displacement of the nucleotide diphosphate–leaving group. By contrast, retaining GTs do not use an enzyme side chain as catalytic base but instead are generally considered to employ a same-side S_N_i-type mechanism where the acceptor hydroxyl nucleophile is deprotonated by the donor β-phosphate oxygen and attacks the anomeric carbon atom of the donor sugar from the same side as the leaving nucleotide (8). While there are rare examples of unusual GTs that presumably employ a double-displacement mechanism (9, 10), in general, the differences in catalytic machinery between inverting and retaining GTs are the location and use of a catalytic base in acceptor deprotonation and the location of the acceptor nucleophile hydroxyl relative to the nucleotide sugar donor (8).

Independent of the catalytic mechanism, GTs can be classified into one of four major folds (A, B, C, and lyso) (1, 8, 11) or variants of known folds (11) based on primary sequence similarity and 3D topology. A vast majority of GTs fall within the GT-A fold, which is characterized by the Rossmann fold–like α/β/α sandwich topology adopted by a diverse class of nucleotide-binding proteins unrelated to GTs (1, 12), but the structural basis for how GTs evolutionarily diverged from other Rossmann fold proteins is not known.

We recently reported a deep evolutionary classification of GT-A fold sequences into 53 (sub)families that broadly fall into nine different clades and identified the core structural features shared among diverse GT-A fold enzymes (13). These core features include two motifs (DxD and xED) involved in catalytic functions as well as an extended network of hydrophobic residues connecting the catalytic and nucleotide-binding sites. While a majority of these conserved hydrophobic residues are present in other Rossmann fold enzymes, a subset of them are unique to GT-A fold enzymes and undergo family specific variations (13). For example, in the B3GNT2 family of GT-A fold enzymes, one of the conserved hydrophobic residues in the F-helix is selectively replaced by a family specific threonine, without any apparent change in the overall structure or fold (14). Furthermore, in a subset of GT-A families such as GT6 and GT8, the GT-A–specific residues are frequently mutated in cancer subtypes (Table S1). However, the structural and functional roles of these natural and disease variations in the core are largely unknown.

Nearly all folded proteins are characterized by hydrophobic residues in the core that contribute to protein folding and stability (15, 16, 17). While most protein cores are optimally packed, in many regulatory and signaling proteins, the core packing is nonoptimal resembling a “nuts-and-bolts” in a jar model (18), in which some core residues are rigid, whereas others are flexible. The overall fitness of a hydrophobic core is determined by energetic favorability of packing interactions (19), and packing efficiency has been correlated with protein dynamics and allosteric functions (20, 21). The nonoptimal packing of the core provides a selective advantage in some proteins, such as protein kinases, which are dynamically assembled during regulation of catalysis. Protein kinases contain an extended hydrophobic network connecting the ATP and substrate-binding lobes, termed the “spines,” which are dynamically assembled during kinase activation (22) and the suboptimal packing of the spine residues enable dynamic regulation of catalytic activity (19, 23, 24). Indeed, malleable cores have been implicated in allosteric regulation or inhibition in other enzyme families as well (25, 26, 27), but the role of conserved core in GT-A evolution and function has not been systematically investigated.

Here using a combination of structural bioinformatics and experimental studies, we investigate the role of conserved hydrophobic core in GT-A structure, function, and evolution. Based on the identification of an ancient phosphate-binding cassette (PBC; (28), Fig. 1) shared by GT-As and other nucleotide-binding proteins, we dissect the hydrophobic core of GT-A enzymes into three categories: residues shared among PBC-containing enzymes, residues shared by Rossmann fold proteins, and residues unique to the GT-A core. We perform an in-depth structural analysis of the GT core–specific residues (residues 156 and 183) connecting the PBC and the αF-helix and find a strong correlation between hydrophobic packing and catalytic mechanism (inverting *versus* retaining). We propose that a dynamic GT-A core provides a selective advantage by enabling new modes of donor- and acceptor-binding functions. Our studies support a model in which the GT-A core evolved progressively through elaboration of an ancient PBC found in diverse nucleotide phosphate–binding proteins. Implications of our findings in the synthetic design of GTs and characterization of oncogenic mutations mapping to the core are discussed.

**Figure 1:**
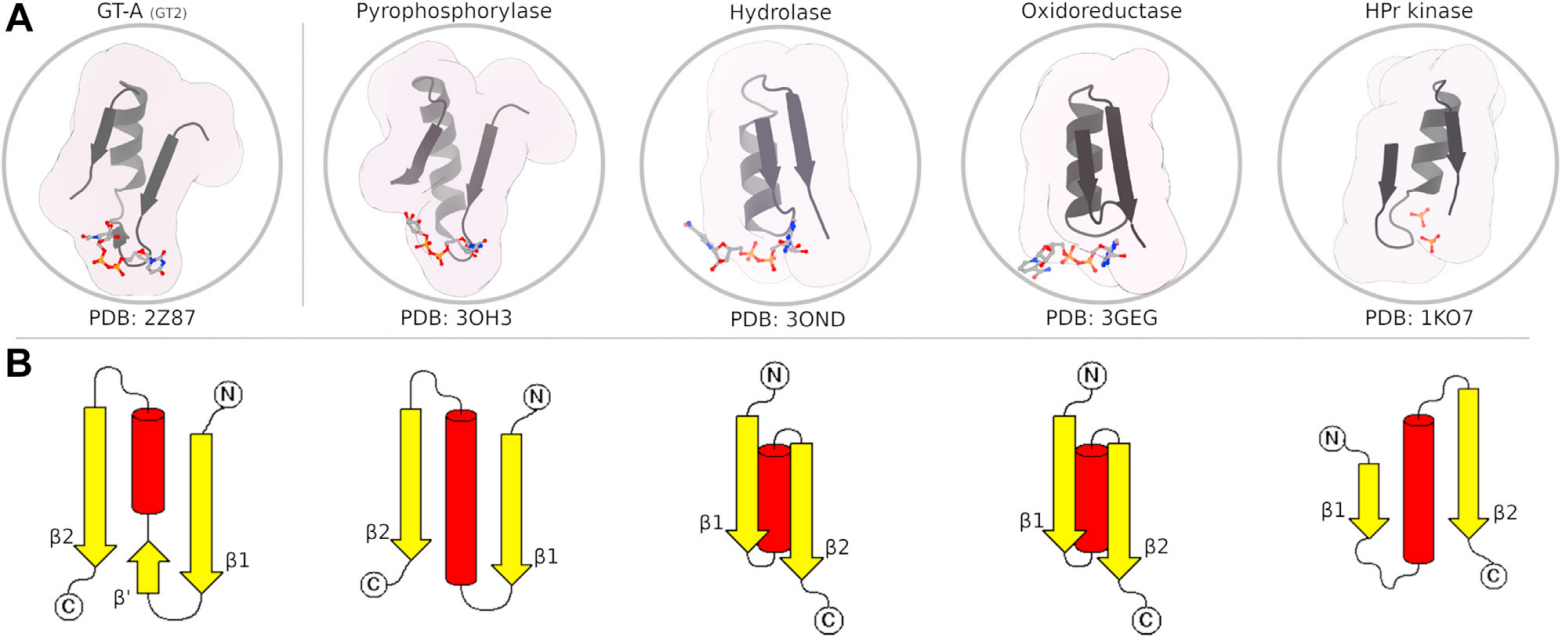
**Structural comparison of the PBC in selected enzyme superfamilies.***A*, cartoon representations of different enzyme superfamilies with a GT-A structure at the *left*, demonstrating superfamily specific variations to a shared ancestral β-α-β phosphate-binding region. *B*, comparison of a subset of GT-A, Rossmann fold, and P-loop NTPase PBC topologies as *cartoons* to show how GT-As structurally differ from most other Rossmann fold enzymes. Many topologies exist to bind the phospho-nucleotide ligand. GT-A, fold-A glycosyltransferase; PBC, phosphate-binding cassette.

## Results

### Delineation of the PBC and modular evolution of the GT-A hydrophobic core

Recently, an ancestral PBC shared among P-loop NTPases and Rossmann fold enzymes was reported (28). This includes several major enzyme superfamilies, such as pyrophosphorylases, oxidoreductases, epimerases, and hydrolases. Now, based on further structural comparisons (see the Experimental procedures section), we extend the presence of this ancestral PBC to GT-As (Fig. 1). We used hidden Markov models (HMMs) from previously published PBC themes (28), which produced significant hits to the PBC of GT-As. Different enzyme families have variable structural topologies of the PBC (28). By performing an all-*versus*-all structural comparison of a representative set of these different PBCs, we identify clusters of PBCs that further support structural and functional similarities between the GT-A PBC and NDP-sugar pyrophosphorylases (Fig. S4). GT-A PBCs closely resemble that of Rossmann pyrophosphorylases in terms of overall topology. Notably, both pyrophosphorylases and GT-As consistently use metal ions to bind the dinucleotide phosphate. Specifically, UDP-sugar pyrophosphorylases bind a UTP donor and sugar-1-phosphate acceptor and catalyze the formation of a UDP-sugar substrate, which is used as a donor substrate for both GT-A and GT-B fold enzymes (1). Structural alignment of the PBCs (using Protein Data Bank [PDB] IDs: 3OH3 and 2Z87) reveals similar PBC topologies for cofactor and nucleotide binding in these two enzymes (Figs. S2–S5). Matching homology from the HMM analysis and the structural alignment suggest a shared ancestry between these two protein families, although the possibility of convergent evolution of a common phosphate-binding mode cannot be ruled out.

GT-A PBCs differ from most other Rossmann fold enzymes and P-loop NTPases by flipping the topological orientation and replacing the glycine-rich loop (located between the β1 sheet and α1 helix) with an additional pseudo beta bridge (β′), shifting the binding site for both the ligand and divalent cation (Figs. 1*B* and S2). Likewise, elaboration of the loop connecting β1 and α1 helix in GT-A through insertion of the metal coordinating DxD motif further contributes to structural and functional divergence of GT-A PBC from other Rossman enzymes (Figs. 1*B* and S2).

**Figure 2:**
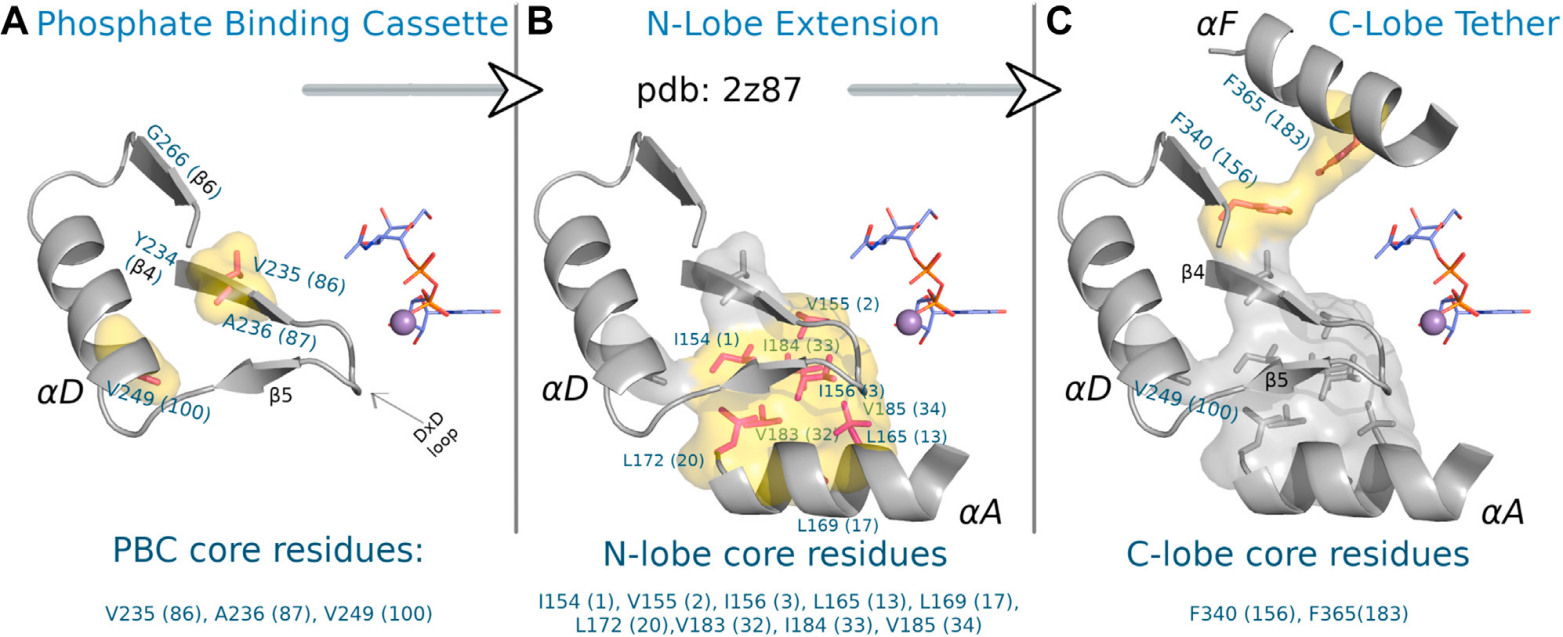
**The GT-A hydrophobic core is separable into three modules over evolutionary time.***A*, structural depiction of the ancestral phosphate-binding cassette (PBC) in GT2 (Protein Data Bank ID: 2Z87), which contains three of the hydrophobic residues of the GT-A core (*surface representation*). *B* and *C*, extension of the hydrophobic core from the PBC, showing the insertion of an N-lobe core, common to all Rossmann fold enzymes, and a GT-A specific C-lobe tether which connects the αF-helix to the PBC. GT-A, fold-A glycosyltransferase.

In GT-As, the PBC corresponds to β4, αD, and β6 (residues Y234 to G266 in PBC; Fig. 2*A*) containing the classic metal-binding DxD motif (1) and a miniature hydrophobic core (Fig. 2*A*). Delineation of the PBC allows us to further dissect the anatomy of the GT-A core into three hierarchical categories based on the depth of conservation of hydrophobic residues. We denote these residues based on the GT2 structure (PDB ID: 2Z87) and the consensus alignment numbering published in a previous study (alignment position indicated parenthetically). Residues present in the PBC include V235 (86), A236 (87), and V249 (100) (Figs. 2*A* and S6). Residues shared by Rossmann fold enzymes include I154 (1), V155 (2), I156 (3), L165 (13), L169 (17), L172 (20), V183 (32), I184 (33), V185 (34), V235 (86), and A236 (87) (Figs. 2*B* and S6); and residues unique to GT-A fold enzymes include V249 (100), F340 (156), and F365 (183) (Figs. 2*C* and S6). Hydrophobic residues shared by Rossmann fold enzymes tether the PBC to the N-lobe (αA-helix), whereas residues unique to GT-A fold enzymes tether the PBC to the αF-helix in the C-lobe. In particular, the GT-A–specific hydrophobic residue in the F-helix (F365; position 183 in Fig. 2*C*) mediate a van der Waals interaction with hydrophobic residues in the PBC (F340 position in Fig. 2*C*) and a backbone hydrogen bond with the catalytic xED-Asp. Because the C-lobe tethering of the PBC is unique to GT-As and represent the most recent addition in GT-A core evolution, we focus on the C-lobe tethering interaction (F340 and F365) in the following sections.

### GT-A–specific extension of the ancestral core is malleable and contributes to conformational flexibility, acceptor recognition, and catalysis

**Figure 3:**
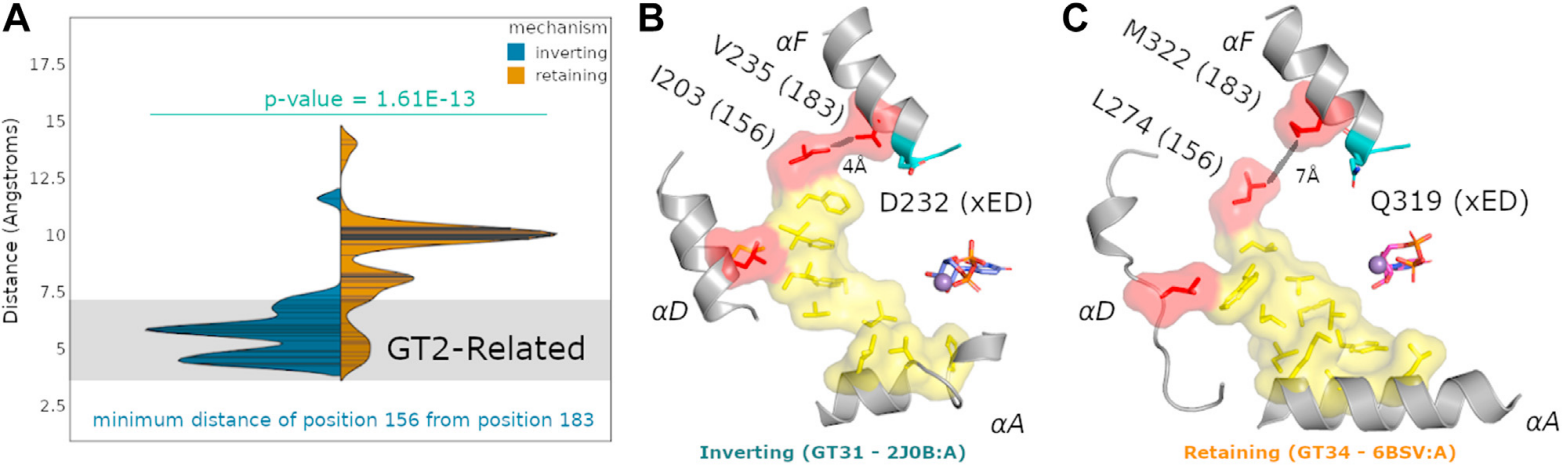
**Structural conservation and variability in the C-lobe tether.***A*, Violin plot of representative GT-A Protein Data Bank structures, separated by mechanism, measuring the minimum distance from hydrophobic core positions 156 and 183, with a line of fit for histogram density showing significant separation between retaining and inverting GT-As (*p* = 1.61E-13). The g*ray bar* indicates the range for a typical hydrophobic contact. Retaining GT-As show a higher variation than inverting GT-As for this region, with most retaining GT-As having a minimum distance between 9 and 10 Å, greater than a hydrophobic contact. Inversely, most inverting GT-As appear to maintain a contact distance of ∼3 to 6 Å, within contact distance. *B* and *C*, structural differences between retaining and inverting GTs, using two representative GT-A structures reveal a separation in most retaining GTs that appears to extend the size of the hydrophobic core. Core residues in *yellow* are conserved across all Rossmann fold enzymes, whereas *red residues* are GT-A specific. Where most inverting cores (*blue*) can directly make contacts in the tether, many retaining GTs have a gap between these conserved residues from packing defects. GT-A, fold-A glycosyltransferase.

We performed a detailed analysis of the structural interactions mediated by tether residues (at positions 156 [F340] and 183 [F365]) in representative crystal structures to investigate their role in GT-A fold structure. Analysis of the contact distances between these residues indicates significant variability in side-chain contact distances (ranging from 4 to 14 Å) across diverse GT-A enzymes. Further analysis of these distances in inverting and retaining enzymes revealed strong correlation between contact distance and catalytic mechanism (*p* = 1.61E-13, using a two-tailed *t* test) (Fig. 3*A*, Supp File 156-183_dist).

In inverting GT-As, the hydrophobic contact distance between 156 and 183 is in the range of 4 to 7 Å, whereas in the majority of retaining GT-As, the median distance between these residues increases significantly, with a normalized maxima around 10 Å. Retaining GT-As form a bimodal distribution, where several retaining GT-As have a contact distance between 4 and 7 Å. We observe these retaining GT-As to appear in clades containing previously phylogenetically classified subfamilies (12) of the large GT2 CAZy family, thus we term these as “GT2 related” (Figs. 3*A* and S7). GT2s are more primordial (12), and as such, we note that retaining enzymes related to GT2 have largely maintained a spacing consistent with the more constrained inverting enzymes. More distant retaining GT-As appear to have a less tightly packed C-lobe tether (Fig. 3, *B* and *C*).

**Figure 4:**
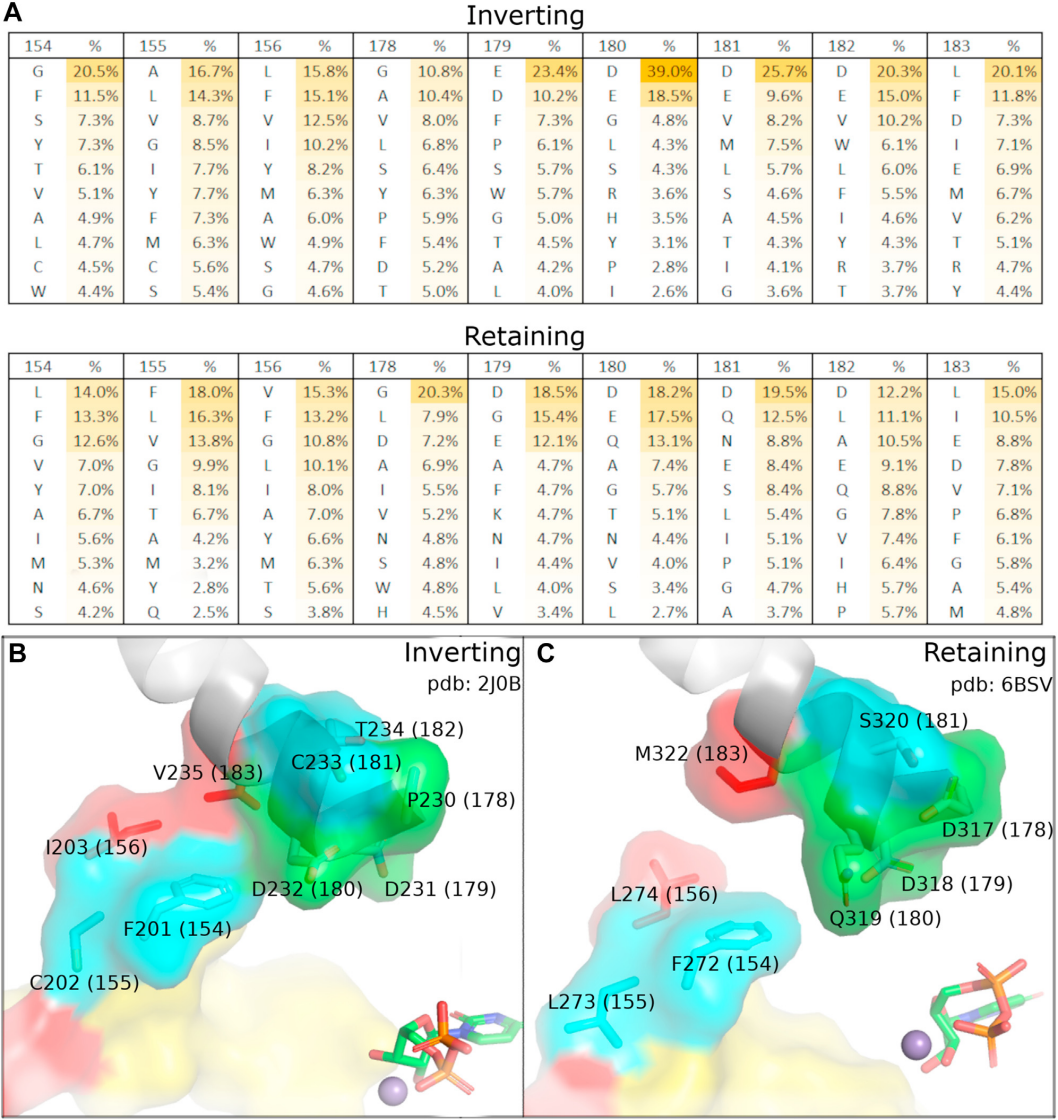
**Amino acid preferences in the C-lobe tether of inverting and retaining enzymes.***A*, array of the top ten residue frequencies from a sequence alignment of inverting and retaining GTs, showing higher conservation and constraints in the C-terminal tether (156, 183) and xED-Asp (180) in inverting GT-As. A full table of these residue frequencies is shown in Table S4. *B* and *C*, a comparison of representative inverting and retaining GT-A core packing in the same orientation, showing that the retaining pocket is less packed, as compared with inverting GT-As. The xED is highlighted in *green*, the C-lobe tether residues are highlighted in *red*, and in *blue* are residues in the logo adjacent to the C-lobe tether. GT, glycosyltransferase; GT-A, glycosyltransferase.

While the catalytic base (xED-Asp) is conserved in inverting GTs, in retaining enzymes, the xED-Asp is often replaced by a glutamine or a glutamate, which shifts the site of catalysis by >2 Å (8), preventing it from being used as a catalytic base. Instead of the xED motif, retaining GTs use the β-phosphate oxygen of the UDP-sugar donor as a catalytic base and perform a dissociative S_N_i-type reaction mechanism (8). To determine whether the loss of constraint on the xED-Asp in retaining enzymes correlates with packing in the C-lobe tether, we analyzed the nature of residues surrounding the tether in primary sequences and 3D structures (Fig. 4). Comparisons of inverting and retaining GTs indicate differences in both xED-Asp position as well as residues involved in C-lobe tether (Fig. 4*A*). We further compare core packing interactions between representative GT-A crystal structures, and note that the retaining GT-As have a less tightly packed tether because of a substitution of a flexible methionine (M322) by a valine (V235), which alters core packing (Figs. 4, *B* and *C* and S3). In a subset of GTs, such as GT15, the hydrophobic tether is replaced by a salt bridge interaction (Fig. S9). Likewise, in B3GNT2 (GT31), a conserved water molecule is involved in the tethering interaction (Fig. S9*E*). The structural and functional implications of these family specific variations are discussed later.

### B3GNT2-specific variations in the C-lobe tether contribute to catalytic activity, stability, and dynamics

**Figure 5:**
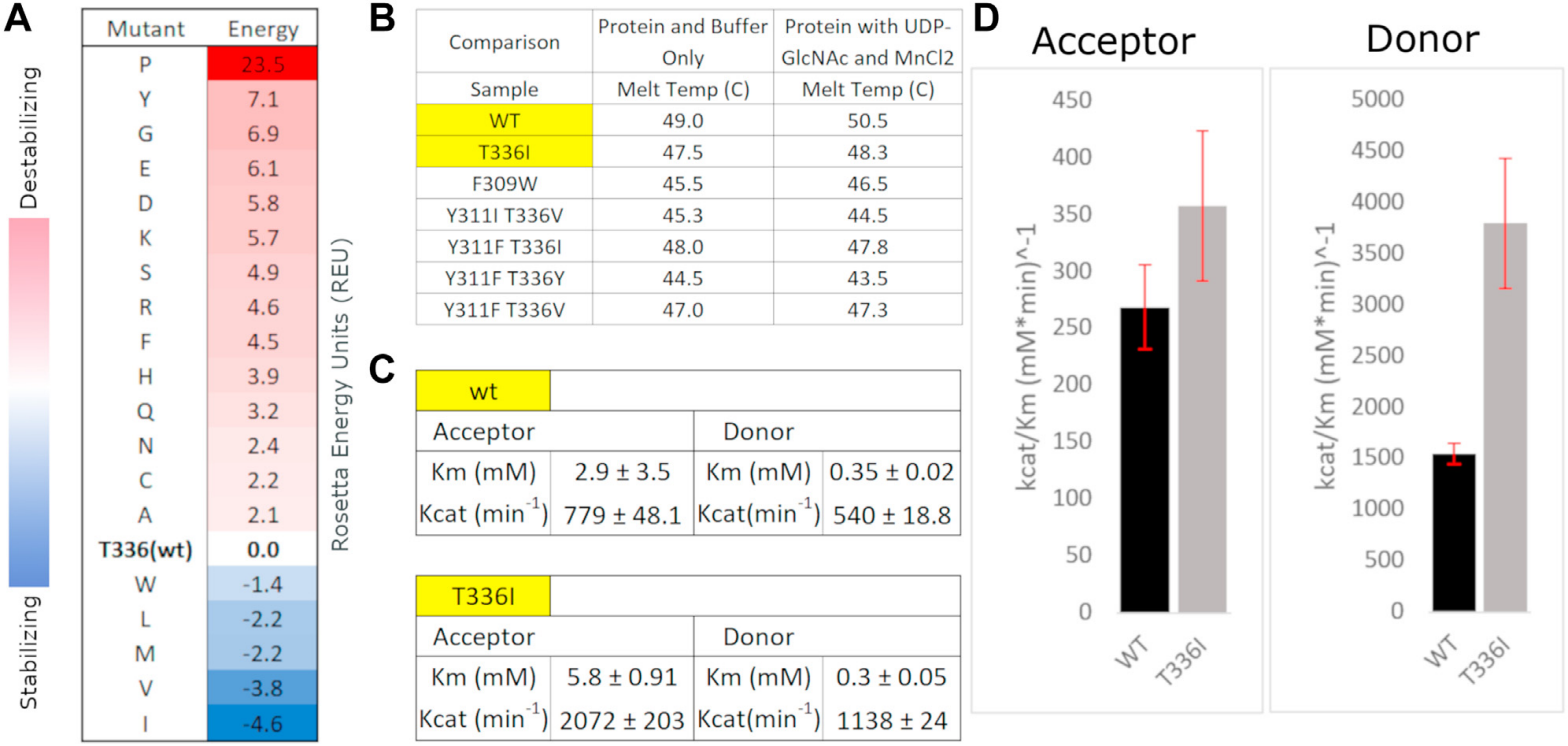
**Computational and experimental screen of B3GNT2-specific variations in the C-lobe tether.***A*, computational mutational screen of the T336 mutants to identify potential stabilizing mutations. *B*, thermostability data of T336I mutant and wt B3GNT2, with all other mutants. *C*, table of kinetic parameters for acceptor and donor saturation in wt and T336I. *D*, kinetic efficiency (*K*_cat_/*K*_m_) of B3GNT2 wt *versus* T336I upon acceptor and donor saturation, demonstrating a 1.3-fold and 2.5-fold increase, respectively, for the T336I relative to wt.

We next investigated the structural and functional implications of B3GNT2-specific variation in the C-lobe tether. In B3GNT2 crystal structures, the threonine (T336) side chain forms van der Waals interactions with hydrophobic residues (F156) in the phosphate-binding module to maintain the C-lobe tether. Also, the small size of the threonine side chain creates internal cavities that are occupied by a water molecule, which coordinate with the hydroxyl group of T336 side chain as well as the xED-Asp. To investigate the structural and functional implications of these B3GNT2-specific variations, we performed a computational and experimental screen of different variants at position 183 (T336). A computational screen using Rosetta predicted a subset of stabilizing and destabilizing mutations (Fig. 5*A*).

With these predicted sets of stabilizing and destabilizing mutations, we then experimentally expressed a subset of single and double mutants (F309W, T336I, Y311I/T336V, Y311F/T336I, Y311F/T336Y, and Y311F/T336V) through recombinant expression in human embryonic kidney 293 cells (14). All the generated mutants expressed at detectable levels and did not impair folding or secretion (Table S3 and Fig. S10). We next examined the thermostability and catalytic activity of these mutants using thermal shift assays and Promega UDP-Glo assays, respectively. The mutants altered thermal stability to varying degrees. While T336I, Y311F/T336I, and Y311F/T336V were partially destabilizing (∼2 °C relative to wt), F309W, Y311I/T336V, and Y311F/T336V were more destabilizing (>4 °C relative to wt) (Fig. 5*B*).

Analysis of the kinetic efficiency (*k*_cat_/*K*_*m*_) of the mutants revealed varying impact on substrate affinity (*K*_*m*_) and turnover (*k*_cat_). In particular, catalytic activity of T336I increases by approximately twofold relative to wt, under acceptor and donor saturation (Fig. 5, *C* and *D* and Table S2). The *K*_*m*_ of T336I increased twofold under acceptor saturation and decreased by 0.15-fold under donor saturation. The catalytic efficiency of T336I increased by 1.3-fold and 2.5-fold under acceptor and donor saturations, respectively (Fig. 5*D* and Table S2). On the other hand, the F309W mutant displayed catalytic efficiency comparable to wt upon acceptor saturation, and a 1.93-fold increase in efficiency upon donor saturation, despite reduced thermostability. The other mutants, generally, displayed decreased catalytic efficiency relative to wt (Table S2).

**Figure 6:**
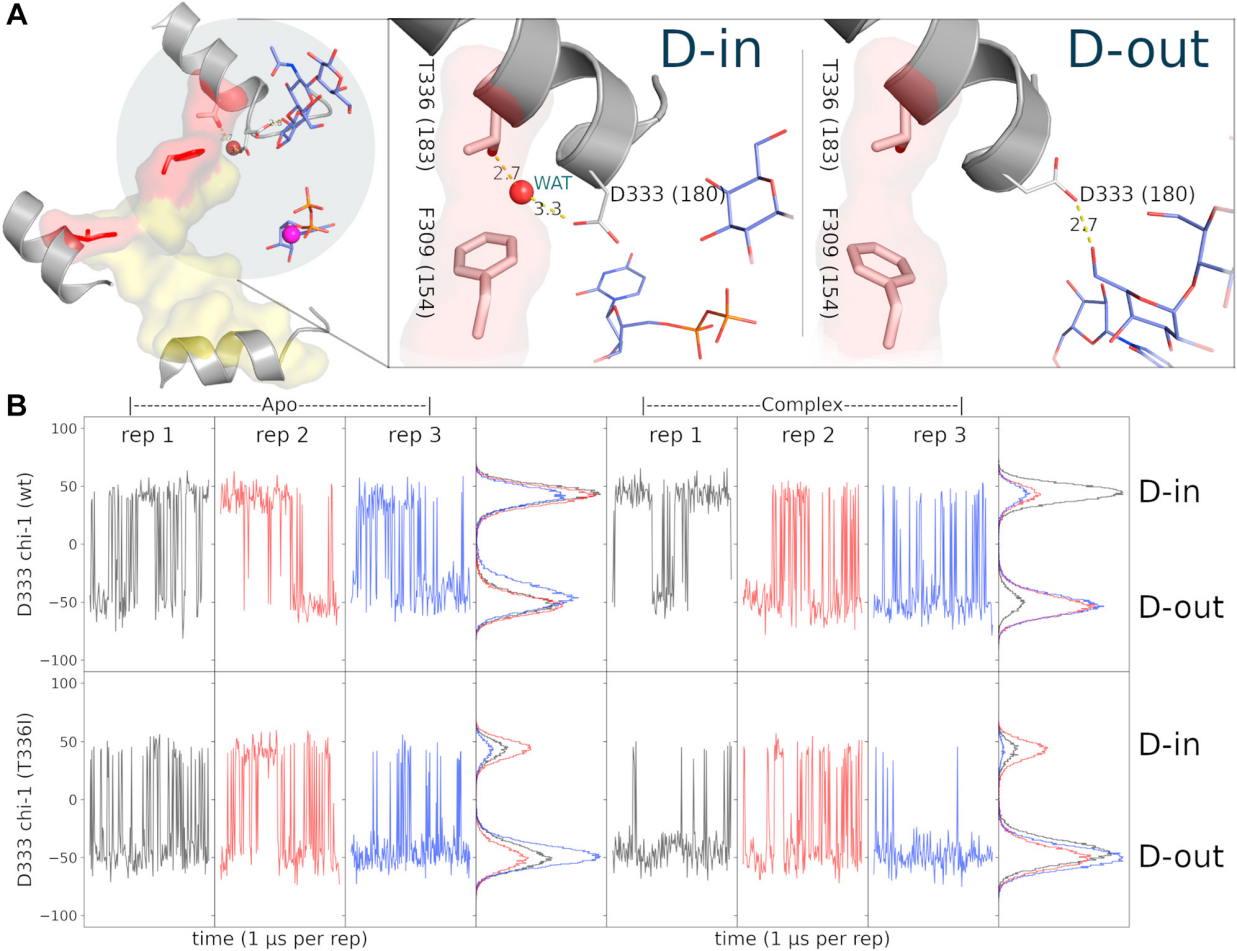
**Molecular dynamics simulations of wt and mutant B3GNT2.***A*, snapshots from an MD simulation of the wt complex, showing two unique conformations of the xED-Asp. The D-in and D-out conformations are termed as such depending on their orientation inward, interacting with the threonine aided through a hydrogen bond interaction with a water molecule, or outward toward the acceptor–donor complex. *B*, 12 MD simulations (three replicates, 1 μs each) demonstrating the conformational shift of mutant T336I to the D-out conformation. Replicates show the dynamic switching between the D-in and D-out conformations over the course of the simulation, with the histograms showing the total ratio of D-in:D-out for each replicate. MD, molecular dynamics.

To investigate the structural basis for the increased activity observed for the T336I mutant, we performed microsecond time-scale molecular dynamics (MD) simulations of wt and mutant B3GNT2 (Fig. 6), focusing on the conformational changes associated with the xED-Asp. In the crystal structure, the xED-Asp (D333) exists in two distinct conformations: D-in and D-out. In the D-in conformation, the xED-Asp is pointing toward the hydrophobic core and forms a water-mediated hydrogen bonding network with T336. In the D-out conformation, the xED-Asp points out toward the acceptor-binding site and forms a hydrogen bond with a hydroxyl group in the acceptor-bound complex where it acts as catalytic base (Fig. 6*A*). In the MD simulations of wt B3GNT2, both these conformations are equally sampled in the apo and acceptor-bound complexes (Fig. 6*B*). However, in the T336I mutant, the xED-Asp is predominantly observed in the D-out conformation. The D-in conformation is not sampled as frequently in the mutant, since the Ile substitution occludes the water-binding site in wt B3GNT2. The shift in the conformational occupancy of the xED-Asp in the acceptor-bound “out” conformation may explain the partial increase in catalytic activity observed for the T336I mutant because the xED-Asp is readily able to deprotonate the acceptor. We further note that in the crystal structure of the closest relative, Manic fringe (PDB ID: 2J0A (29)), which contains a valine in place of the threonine, the xED-Asp adopts the D-out conformation in the crystal structure. Indeed, MD simulation with a valine mutant also demonstrates a preference for the D-out conformation (Fig. S11). Finally, we note that protonation of the xED-Asp also alters conformational dynamics (Figs. S12 and S13) primarily through changes in the chi-2 dihedral, as noted in other systems (30, 31). Based on these MD simulations, we hypothesize that changes in p*K*a may influence B3GNT2 catalytic activity. Together, our simulations provide additional support for our hypothesis that GT-A fold catalytic activities and mechanisms can be fine-tuned through mutations in the GT-A–specific C-lobe tether.

## Discussion

### A proposed modular evolution of GT-As

**Figure 7:**
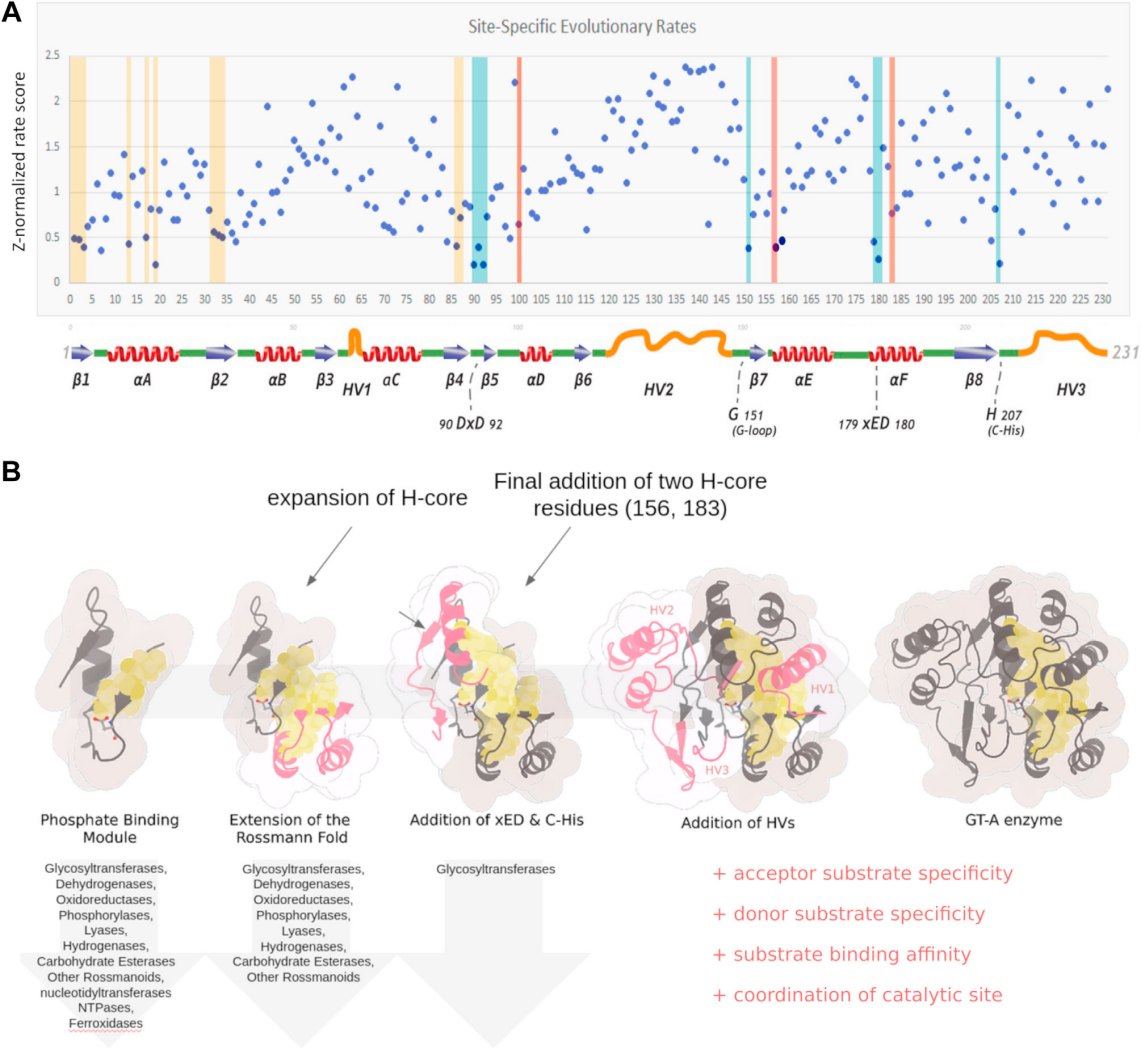
**Modular evolution of GT-As.***A*, site-specific rate conservation of each residue of the 231 aligned positions. *Dots in yellow bars* reflect hydrophobic residues common to all Rossmann fold enzymes. *Dots in blue bars* reflect functional motifs, including DxD, G-loop, xED, and the C-His. *Dots in red bars* are GT-A–specific residues of the hydrophobic core. *B*, model of the evolutionary progression of fold A glycosyltransferases. Beginning from the elementary phosphate-binding cassette, GT-As gained a Rossmann fold that extended the hydrophobic core. Following this, various GT-As make use of the xED motif as a catalytic base, the presence of this motif correlates with mechanistic variations. Finally, family specific hypervariable regions are introduced to further regulate GT-A function. New additions in *pink*. GT-A, fold-A glycosyltransferase.

In our previous study comparing GT-A fold enzymes from diverse organisms, we identified a conserved hydrophobic core under strong selective pressure, as reflected by the low evolutionary rates of these residues among the 231 aligned positions in the GT-A catalytic domain (Figs. 7*A* and S14). Here, we further dissect the anatomy of the core based on a broader analysis of diverse nucleotide-binding Rossman fold enzymes. Our studies reveal three distinct GT-A core modules added over evolutionary time (Fig. 7*B*) that are further embellished by family specific hypervariable regions. The first module is contained within an ancestral PBC, common to many nucleotide phosphate–binding enzymes. Ancestral phosphate-binding enzymes embellished upon this core to maintain its phosphate-binding function while resulting in the functionally diverse superfamilies that exist today. This core serves a similar function in GT-As by conserving motifs (specifically, the DXD motif) that are directly involved in binding the phosphate moiety of the donor substrate. GT-As, along with many other enzyme families, build upon this PBC to form the Rossmann fold, which binds a diverse array of cofactors including nucleotide sugars (32). We note different topological orientations of the PBC in enzyme families, even within the P-loop NTPases (28). However, the similarities between pyrophosphorylases and GT-As, in terms of shared PBC topologies, nucleotide, and divalent cation binding, suggests either convergent evolution, or a common ancestor connecting these enzyme families.

Extant GT-A fold enzymes extended the phosphate-binding module through addition of a unique C-terminal extension of the hydrophobic core, facilitated by the residues 156 and 183 (F340 and F365 in GT2), which tethers the F-helix and xED catalytic base to the PBC. The tether aids in positioning the catalytic base residue for inverting GTs critical for their S_N_2 displacement mechanism (8). Among retaining GTs, the tether to the F-helix and positioning of the xED motif is maintained, but since catalytic base function for most retaining enzymes is accomplished by the β-phosphate oxygen of the sugar nucleotide donor (8), selective pressure for maintaining the position of the catalytic base relative to the sugar donor is no longer needed. As a result, residues flanking the xED in retaining GT-As may be more malleable and likely to mutate, allowing these GT-As to sample new acceptor interactions and other functions, resulting in increased tethering variation.

We previously proposed that inverting and retaining mechanisms evolved multiple independent times during GT-A enzyme evolution by generating a phylogenetic tree of diverse GT-A fold enzymes (12). Here, we show that variations in the C-lobe tether may have contributed to this multiple independent evolution by altering core packing and xED-base positioning for either an associative mechanism or a dissociative mechanism. Consistent with this view, retaining GTs, mostly the ones that are further away from inverting families in the phylogenetic tree (GT2 unrelated, Fig. S7), tend to elongate the C-lobe tether with distances around 9 to 10 Å, often even accommodating extra residues between these positions (Figs. 3*C* and S8). In contrast, inverting GTs and GT2-related retaining GT-As have a tightly packed tether with inter-residue distances of around 3 to 4 and 5 to 7 Å, respectively. Multiple GTs show variability in this tether, even going so far as to change the packing interactions from van der Waals to salt bridges (Fig. S9). We note that the retaining GTs, GT55 (mannosyl-3-phosphoglycerate synthase) and GT15 (glycolipid 2-α-mannosyltransferase) that are divergent (located in different branches of the tree), have a salt-bridge tether in common, suggesting that this variation may not just be structural but may have a functional role. Notably, both GTs are mannosyltransferases that catalyze transfer to unique acceptors; GT55 to a phosphate-linked glycerate acceptor and GT15 to a glycolipid (33, 34). These two mannosyltransferases, accommodating different acceptor substrates, may suggest a convergent evolution of this tether and one of multiple solutions that influences accommodation of a vast diversity of acceptor–donor complexes. Thus, variability and malleability of the C-lobe tether provides the structural framework for multiple independent paths for evolutionary interconversion of retaining and inverting mechanisms on a common fold.

The regulatory functions of a flexible hydrophobic core have been well articulated in large protein superfamilies such as kinases (35). Here, through computationally aided mutational analyses and MD simulations of the C-lobe tether in B3GNT2, we demonstrate that this GT-A–specific extension contributes to the functional stability of the enzyme. Introduction of the more canonical hydrophobic packing in the C-lobe tether favored the D-out conformation of the xED-Asp. This D-out conformation was also observed in the native crystal structures of a related GT31 enzyme, Manic fringe (29, 36), which has a valine in place of B3GNT2’s threonine. By changing the conformational occupancy of the catalytic base, wt B3GNT2 may illustrate an evolutionary mechanism to fine-tune catalytic activity. Accumulation of such mutations provides the basis for large-scale transitions in enzyme function during evolution (18, 19, 37).

An analysis of cancer variants cataloged in The Cancer Genome Atlas and COSMIC (the Catalogue Of Somatic Mutations In Cancer) reveals nearly 420 nonsynonymous mutations mapping to the GT-A hydrophobic core, 47 of which map to the C-lobe tether (Table S1 and Fig. S15). Most of these mutations are predominantly located in the GT8 subfamilies, such as GT8-LARGE, and change the size or biochemical properties of the hydrophobic residues. Investigating how these oncogenic mutations impact GT structure and regulation will further illuminate the functions of the understudied GT-A core in disease states. The ability to switch substrate preferences and control enzyme kinetics through malleable cores could mark the fine margins to ensure proper glycosyl transfer. As such, understanding the intricate mechanisms that guide the activity of these diverse enzyme families allows us to engineer new regulatory functions, and we believe that the identification of the critical rheostat functions played by the hydrophobic core could pave the way for rational design and engineering of GTs with new functional properties.

## Experimental procedures

### Hydrophobic core distance plots

To get minimum distances for each aligned hydrophobic residue in each PDB, we first split each chain from 470 GT crystal structures taken from the CAZy database into 972 PDBs. We then wrote a script using the Biopython module (38) to measure the minimum distances of each aligned hydrophobic position amongst each other. We only used structures with a resolution under 2.5 Å. We generated csv files of these positions and minimum atomic distance values, generating plots of each residue distance, as well as all-*versus*-all median distances for each hydrophobic core position (Fig. S16). With this table, we were able to categorize these GTs by (sub)family and mechanism and generate plots of the extended core. To avoid bias by PDBs that are overrepresented in the available GT-A structures, we performed a CD-HIT query on all available PDB sequences at 90% sequence similarity to generate a diverse and representative set of PDBs for structural informatics studies.

### Rosetta modeling

Structural minimization and loop modification were performed, in preparation for MD simulations, using Rosetta’s kinematic loop generation protocol (23). Structures underwent 10,000 cycles of minimization to prevent atomic clashes *in silico*.

### Oncogenic variant analyses

Full-length GT-A sequences were mined from The Cancer Genome Atlas (39) and COSMIC databases. These sequences were mapped to previously published GT-A profiles (10). Mutations falling at hydrophobic core positions were collected, and duplicate counts were pruned based on patient and sample IDs to get a final count.

### Mutational analyses

For the B3GNT2 structure, we computed mutations for every amino acid for the equivalent positions at 154 and 183 (F309 and T336 in B3GNT2 [PDB ID: 6WMN]). These mutations were performed using the cartesian DDG protocol (40, 41), with three replicates. Rosetta energies were averaged to produce the table of energy values in Table S2. From this table, we picked, based on Rosetta energy scores, sets of stabilizing and destabilizing mutations. A critical caveat to note is that the Rosetta energy score only gives a relative indication of whether a structure is stabilizing or destabilizing. This method does not consider backbone rearrangement upon a mutation that changes packing; thus, the score does not always reflect *in vitro* data. Nevertheless, these scores provide an adequate basis for selecting mutations.

### Mutant expression and purification

The B3GNT2 wt construct was generated as previously described (14). Site-directed mutagenesis was performed using the Q5 Site-Directed Mutagenesis Kit (New England Biolabs) to generate the six mutant B3GNT2 samples. Recombinant B3GNT2 and mutants were generated by transfection of 100 ml cultures of FreeStyle 293-F cells (Thermo Fisher Scientific) as previously described (14). Six days after transfection, the samples were harvested using centrifugation, and enzyme in the culture supernatant was purified by Ni^2+^–nitrilotriacetic acid chromatography. Final samples were buffer exchanged into 25 mM Hepes and 300 mM NaCl, pH 7.5, concentrated by ultrafiltration, and protein concentration was determined using GFP-fluorescence and UV absorbance using a Nanodrop spectrophotometer. The samples were buffer exchanged into 25 mM Hepes and 300 mM NaCl and verified for purity and length using SDS-PAGE gels.

### Sequence analysis

Sequence logos were generated using WebLogo 3.0 and GTXplorer (42, 43), using sequence alignments generated in our previous article (13). We performed the structure-based sequence alignment using PROMALS3D and visualized the sequence alignment using ESPript3 (44, 45). The secondary structure representation in the alignment was generated using data from the DSSP output (46) on the GT2 crystal structure (PDB ID: 2Z87). Calculation of deletions was performed by counting the percentage of gaps in a position across the sequence alignment (Fig. S17).

### HMM analysis

Utilizing HMMs produced from Ref. (47), we ran searches across available GT-A sequences using HMMsearch (48). These searches detected significant similarities in the PBC of P-loop NTPases and a subset of Rossmann fold enzymes, including GT-As. We then took a broad number of the PBCs from the published HMMs along with a set of representative PBCs from GT-As and pyrophosphorylases and performed an all-*versus*-all structural comparison using the TMalign algorithm (49). These RMSDs were then used in a network graph in Cytoscape (National Resource for Network Biology) (50), where nodes represent each PDB and edges represent the RMSD similarity between each node. We used an edge-weighted spring embedded layout to organize the nodes into clusters of closely related proteins. We used a cutoff filter of 2.5 Å to remove the noise of distant connections. This resulted in clusters of closely related proteins, placing UDP-sugar pyrophosphorylases and GT-As next to each other.

### Dihedral analyses

Python code was written for analyzing dihedral angles of residues in PBDs and MD frames (Figs. 5, S11–S13 and S18). This code can be found in the GitHub link in the Data availability section.

### Kinetics

Promega UDP-Glo GT assays were used to analyze the B3GNT2 kinetic parameters as previously described (14). Reactions were performed in a buffer containing 100 mM Hepes, pH 7, 2 mM MnCl_2_, and 1 mg/ml bovine serum albulin in 10 μl reactions using varied concentrations of lacto-N-neotetraose (0.3125–5 mM) as acceptor and UDP-GlcNAc (0.0625–1 mM) as donor to determine the *K*_*M*_ and *k*_cat_ values for wt and mutant B3GNT2 (Table S2 and Fig. S19). Enzyme input varied from 0.156 ng for wt B3GNT2 to 10 ng for severely destabilizing mutations, and each sample was run in biological duplicates.

### MD

Multiple MD simulations were run on the B3GNT2 crystal structures (PDB IDs: 6WMN and 6WMO). We first performed loop modeling using the Kinematic Loop Modeling Protocol in Rosetta to address any missing regions in the structure and then minimized the structure to avoid steric clashes (51). Long time-scale unbiased MD simulations were performed on B3GNT2 at the microsecond level, with two replicates (each 1 μs long). All MD simulations used the Amber99SB-ILDN force field, commonly used for long time-scale protein simulations, along with the GLYCAM06 force field for glycan parameterization (52, 53, 54). Long-range electrostatics were calculated *via* particle mesh Ewald algorithms. All simulations used the TIP3P water model (55). Energy minimization was run for a maximum of 10,000 cycles, performed using the steepest-descent algorithm, followed by the conjugate-gradient algorithm. The system was heated from 0 K to a temperature of 300 K. MD analyses were facilitated in python using the MDAnalysis module (56). After two equilibration steps that lasted 50 ps, microsecond-long simulations were run at a 2 fs timestep.

### Single-molecule charge calculations

We derived the protocol for parameterization of the UDP-donor substrate for the GTs from the GLYCAM force-field article (53). *Ab initio* QM was performed using Gaussian16 to optimize the donor ligand at the HF/6-31G∗ level. We then calculated the charge of the compound using antechamber. The electric charge of the aglycon was previously calculated to be −0.194 au. These parameters were then used to generate ligand input files for use with MD simulations.

### Molecular modeling

The structures were visualized and analyzed in Schrodinger PyMOL 2.0. Structural alignments were performed in PyMOL 2.0 using the cealign algorithm (57). Cartoon models of these structures were created using The Protein Imager (58) to aesthetically portray these structures, after alignment in PyMOL 2.0.

### Site-specific relative evolutionary rate conservation

To produce a normalized conservation value for each aligned position, we used a previously generated alignment, published in our previous article (13), as input into the program Rate4Site (59). This software employs an empirical Bayesian method to calculate a neighbor-joining tree with maximum likelihood distances to output a relative conservation score at each site.

### Thermal shift

ThermoFluor assays were performed in 96-well PCR plates in duplicates with each well containing 45 μl of GFP-tagged protein in the desired buffer at a concentration of 2 μM. The buffer consisted of 25 mM Hepes, 300 mM NaCl, pH 7.5, with 5 μl of 100× SYPRO Orange (Thermo Fisher Scientific). After a 15 min preincubation at room temperature, a melt curve program was run on a Bio-Rad CFX96 machine using a 50 μl total sample volume, from 25 to 95 °C, with a ramp speed of 1 °C/min. The B3GNT2 melt curve was observed in the 40 to 70 °C temperature range based on an increase in SYPRO Orange fluorescence, whereas the GFP fusion tag exhibited an additional melt curve at ∼88 °C.

### AlphaFold2 models

AlphaFold2 produced several previously unknown GT-A structures (60). For subfamilies not found in the AlphaFold2 database, we ran AlphaFold2 on a supercomputer cluster to produce models. After mapping these sequences to known profiles, as described in our previous article (13), we wrote a python script to map alignment positions to these structural models and then visualized the hydrophobic core positions in PyMOL 2.0.

## Data availability

The code and python notebooks used to generate these data analyses are available on https://www.github.com/esbgkannan/GTA_PBC_Core_analysis. The datasets can be found on https://www.dropbox.com/sh/ov93y3z73qgd8th/AADJ5sKuN33tjedn_gwigeI0a?dl=0.

## Supporting information

This article contains supporting information (61, 62, 63).

## Conflict of interest

The authors declare that they have no conflicts of interest with the contents of this article.

## Supporting information

Supplemental Figures S1–S19 and Tables S1–S4

Supp File 156-183_dist
