# Supplementary material for: Modularity of the hydrophobic core and evolution of functional diversity in fold A glycosyltransferases
Source: J Biol Chem. 2022 Jun 30;298(8):102212. doi: 10.1016/j.jbc.2022.102212 (PMC9364030; doi:10.1016/j.jbc.2022.102212)
Supplement: Supplemental Figures S1–S19 and Tables S1–S4 [file mmc1.pdf]

Supporting Information

Supplementary Table 1

Positions	Consensus	Mutants	Counts	Most Common Cancer types (>2 counts)
Position 17	L	I, V, R, M, F, G, S	35	Large Intestine (7), Esophagus (3), Ovary (3), Skin (3)
Position 33	I	I, Y, H, V, L, Q, G, R, K T	54	Upper aerodigestive tract (23), Lung (5), Skin (4), Thyroid (4), Urinary (4), Large Intestine (3), Uterus (3)
Position 34	V	L, I, H, F, Y, Q, T, K, E, N, S, G, P	33	Large Intestine, Endometrium, Liver, Lung, Uterus, Cervix, Skin, Stomach
Position 86	V	L, I, F, V, Y, G, T, S	48	Uterus (8), Kidney (6), Large Intestine (6), Endometrium (5), Stomach (5)
Position 156	F	L, I, Y, V, G, R, S	22	Large Intestine (7)
Position 183	L	I, F, M, R, V, D, S, T, P, Q	25	Large Intestine (5), Stomach (4), Uterus (4), Endometrium (3), Skin (3)

Table S1: Oncogenic mutations observed at the hydrophobic core positions. Mutations were retrieved from TCGA and COSMIC databases and mapped to individual positions in the GT-A catalytic domain using curated alignments published previously (13). Position 33 is a mutation hotspot with variants observed in multipel cancer types.

Supplementary Table 2

Kinetics	Acceptor				Donor			
	Km (mM)	Kcat (min^-1)	Specificity Constant (mM^-1*min^-1)	Mutant (Kcat/Km)/ WT(Kcat/Km)	Km (mM)	Kcat (min^-1)	Specificity Constant (mM^-1*min^-1)	Mutant (Kcat/Km)/ WT(Kcat/Km)
**WT**	2.9 ± 0.36	779 ± 48.1	269 ± 37	1.00	0.35 ± 0.02	540 ± 18.8	1543 ± 103	1.00
**T336I**	5.8 ± 0.91	2072 ±203	357 ± 66	1.33	0.3 ± 0.05	1138 ± 24	3793 ± 637	2.46
**F309W**	5.1 ± 0.49	776 ± 45	152 ± 23	0.57	0.13 ± 0.03	338 ± 24	2985 ± 876	1.93
**Y311I T336V**	8.7 ± 7.8	458 ± 290	53 ± 81	0.20	0.33 ± 0.07	240 ± 23	727 ± 237	0.47
**Y311F T336I**	2.0 ± 0.82	375 ± 69	191 ± 115	0.71	0.6 ± 0.14	672 ± 79	1120 ± 392	0.73
**Y311F T336Y**	4.6 ± 1.2	26 ± 4.1	6 ± 2	0.02	2.3 ± 1.2	53 ± 21	23 ± 21	0.01
**Y311F T336Val**	7.2 ± 1.8	1474 ± 241	204 ± 83	0.76	0.3 ± 0.04	387 ± 25	1290 ± 256	0.84

Table S2: Table of kinetics data of all mutated variants, both single and double mutants, compared to WT.

Supplementary Table 3

Sample	Cell + Media (FLU)	Media Only (FLU)	Protein concentration (mg/L)
**wt**	784.0	655.0	50.0
**T336I**	921.0	786.0	60.0
**F309W**	962.0	807.0	61.6
**Y311F, T336I**	1572.5	1218.0	93.0
**Y311I, T336V**	1345.0	1078.0	82.3
**Y311F, T336V**	1350.5	1039.5	79.4
**Y311F, T336Y**	1287.0	911.0	69.5

Table S3: Fluorescence data (FLU) reported for each B3GNT2 variant expressed in HEK293 cells, along with protein concentration. Corresponding table of data for Figure S10.

Supplementary Table 4

Inverting

154	%	155	%	156	%	178	%	179	%	180	%	181	%	182	%	183	%
A	4.9%	A	16.7%	A	6.0%	A	10.4%	A	4.2%	A	2.4%	A	4.5%	A	2.0%	A	4.2%
C	4.5%	C	5.6%	C	1.6%	C	1.2%	C	0.3%	C	1.0%	C	1.9%	C	2.2%	C	0.8%
D	0.7%	D	1.6%	D	0.9%	D	5.2%	D	10.2%	D	39.0%	D	25.7%	D	20.3%	D	7.3%
E	0.9%	E	0.7%	E	1.1%	E	3.1%	E	23.4%	E	18.5%	E	9.6%	E	15.0%	E	6.9%
F	11.5%	F	7.3%	F	15.1%	F	5.4%	F	7.3%	F	1.4%	F	2.7%	F	5.5%	F	11.8%
G	20.5%	G	8.5%	G	4.6%	G	10.8%	G	5.0%	G	4.8%	G	3.6%	G	2.9%	G	3.0%
H	3.1%	H	1.2%	H	2.1%	H	2.6%	H	3.6%	H	3.5%	H	1.7%	H	2.2%	H	0.5%
I	3.5%	I	7.7%	I	10.2%	I	4.3%	I	2.6%	I	2.6%	I	4.1%	I	4.6%	I	7.1%
K	3.0%	K	1.6%	K	1.6%	K	2.8%	K	1.9%	K	1.0%	K	1.2%	K	1.0%	K	1.9%
L	4.7%	L	14.3%	L	15.8%	L	6.8%	L	4.0%	L	4.3%	L	5.7%	L	6.0%	L	20.1%
M	2.8%	M	6.3%	M	6.3%	M	3.6%	M	1.7%	M	0.2%	M	7.5%	M	1.4%	M	6.7%
N	3.8%	N	1.7%	N	0.9%	N	4.0%	N	2.8%	N	1.2%	N	2.7%	N	1.9%	N	0.7%
P	1.7%	P	1.4%	P	0.2%	P	5.9%	P	6.1%	P	2.8%	P	1.9%	P	2.9%	P	1.3%
Q	0.9%	Q	0.5%	Q	0.2%	Q	2.8%	Q	3.5%	Q	2.1%	Q	2.1%	Q	1.0%	Q	1.0%
R	3.1%	R	0.3%	R	1.4%	R	3.5%	R	2.2%	R	3.6%	R	3.3%	R	3.7%	R	4.7%
S	7.3%	S	5.4%	S	4.7%	S	6.4%	S	5.7%	S	4.3%	S	4.6%	S	3.1%	S	3.0%
T	6.1%	T	1.9%	T	1.9%	T	5.0%	T	4.5%	T	1.2%	T	4.3%	T	3.7%	T	5.1%
V	5.1%	V	8.7%	V	12.5%	V	8.0%	V	2.2%	V	1.9%	V	8.2%	V	10.2%	V	6.2%
W	4.4%	W	1.0%	W	4.9%	W	1.9%	W	5.7%	W	1.0%	W	2.1%	W	6.1%	W	3.2%
Y	7.3%	Y	7.7%	Y	8.2%	Y	6.3%	Y	3.1%	Y	3.1%	Y	2.7%	Y	4.3%	Y	4.4%

Retaining

154	%	155	%	156	%	178	%	179	%	180	%	181	%	182	%	183	%
A	6.7%	A	4.2%	A	7.0%	A	6.9%	A	4.7%	A	7.4%	A	3.7%	A	10.5%	A	5.4%
C	0.4%	C	1.4%	C	1.7%	C	3.1%	C	3.0%	C	1.7%	C	1.3%	C	0.0%	C	0.0%
D	1.4%	D	0.7%	D	0.7%	D	7.2%	D	18.5%	D	18.2%	D	19.5%	D	12.2%	D	7.8%
E	3.9%	E	1.8%	E	0.3%	E	2.7%	E	12.1%	E	17.5%	E	8.4%	E	9.1%	E	8.8%
F	13.3%	F	18.0%	F	13.2%	F	2.7%	F	4.7%	F	2.4%	F	2.0%	F	4.4%	F	6.1%
G	12.6%	G	9.9%	G	10.8%	G	20.3%	G	15.4%	G	5.7%	G	4.7%	G	7.8%	G	5.8%
H	3.5%	H	2.1%	H	0.7%	H	4.5%	H	3.0%	H	2.4%	H	1.7%	H	5.7%	H	3.7%
I	5.6%	I	8.1%	I	8.0%	I	5.5%	I	4.4%	I	2.4%	I	5.1%	I	6.4%	I	10.5%
K	3.9%	K	1.4%	K	1.0%	K	3.8%	K	4.7%	K	2.4%	K	3.4%	K	1.7%	K	2.0%
L	14.0%	L	16.3%	L	10.1%	L	7.9%	L	4.0%	L	2.7%	L	5.4%	L	11.1%	L	15.0%
M	5.3%	M	3.2%	M	6.3%	M	2.4%	M	1.3%	M	1.7%	M	0.7%	M	1.4%	M	4.8%
N	4.6%	N	1.8%	N	2.4%	N	4.8%	N	4.7%	N	4.4%	N	8.8%	N	1.7%	N	2.0%
P	2.1%	P	0.4%	P	1.7%	P	2.7%	P	2.7%	P	0.3%	P	5.1%	P	5.7%	P	6.8%
Q	0.4%	Q	2.5%	Q	0.3%	Q	1.0%	Q	2.3%	Q	13.1%	Q	12.5%	Q	8.8%	Q	4.1%
R	1.1%	R	2.5%	R	3.5%	R	3.4%	R	2.3%	R	1.7%	R	2.0%	R	0.7%	R	3.4%
S	4.2%	S	1.8%	S	3.8%	S	4.8%	S	2.0%	S	3.4%	S	8.4%	S	2.0%	S	3.4%
T	2.8%	T	6.7%	T	5.6%	T	3.1%	T	0.7%	T	5.1%	T	3.0%	T	1.0%	T	1.4%
V	7.0%	V	13.8%	V	15.3%	V	5.2%	V	3.4%	V	4.0%	V	1.7%	V	7.4%	V	7.1%
W	0.4%	W	0.7%	W	0.7%	W	4.8%	W	3.0%	W	1.7%	W	0.3%	W	0.0%	W	0.0%
Y	7.0%	Y	2.8%	Y	6.6%	Y	3.1%	Y	3.0%	Y	2.0%	Y	2.4%	Y	2.4%	Y	1.7%

Table S4: Full table of residues conserved at each of the core aligned positions in inverting and retaining enzymes. Degree of conservation is demarcated by intensity of orange and previously published alignments (13) were used for estimating conservation.

Supplementary Figure 1

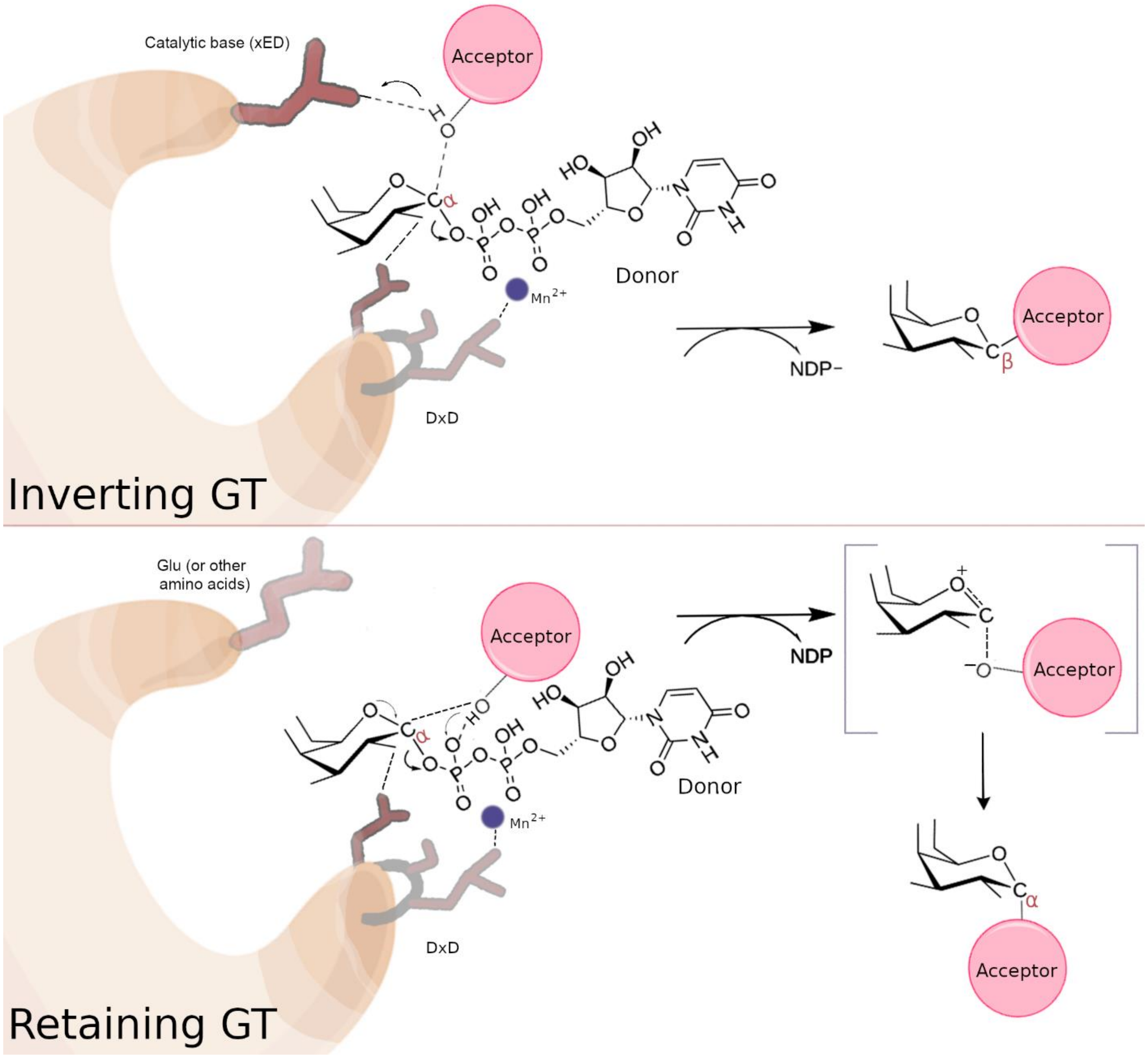

Figure S1: Cartoon representation of the inverting and retaining mechanisms. Inverting mechanisms use the catalytic base (xED) to deprotonate the acceptor, resulting in an inversion of the anomeric linkage (alpha to beta or beta to alpha). Retaining enzymes generally proceed via a front-facing attack (S_ni), where the donor deprotonates the acceptor hydroxyl, leading to retention of the linkage (alpha to alpha or beta to beta).

Supplementary Figure 2

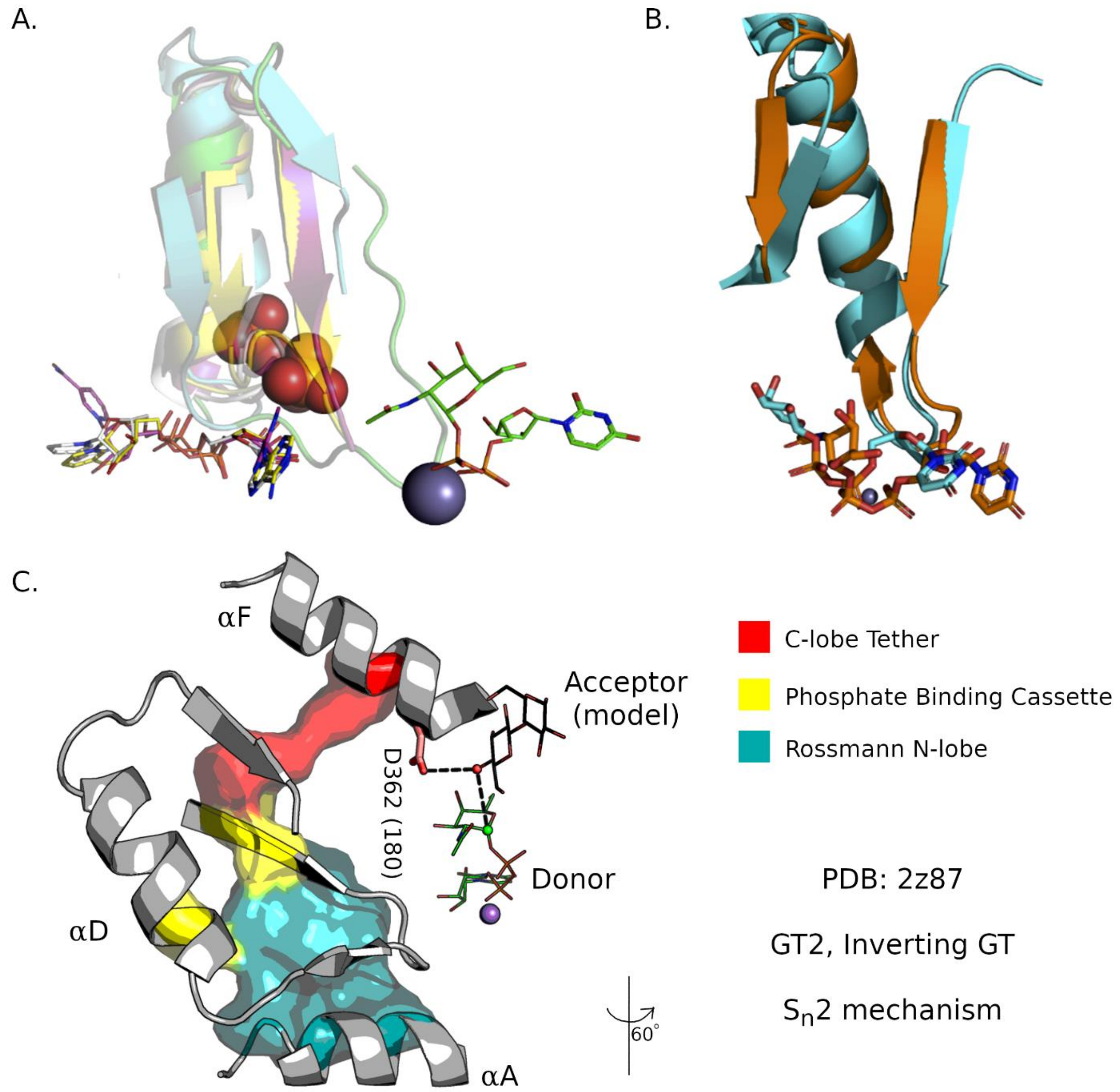

Figure S2: A) Cartoon representations of different enzyme superfamilies structurally super-positioned with a GT-A structure (GT2) (in green), along each superfamily's respective nucleotide phosphate substrate. B) Cartoon representations of only GT2 (pdb: 2Z87, orange) and a UDP-Sugar pyrophosphorylase (pdb: 3OH3, blue) as seen in figure 1. They are structurally super-positioned with the respective UDP-sugar ligands bound, showing that are positioned in the same location and orientation. C) Visualization of the binding mode of GT2 in context of each module of the hydrophobic core, using an acceptor modeled from a related GT2 structure (pdb: 7D5K).

Supplementary Figure 3

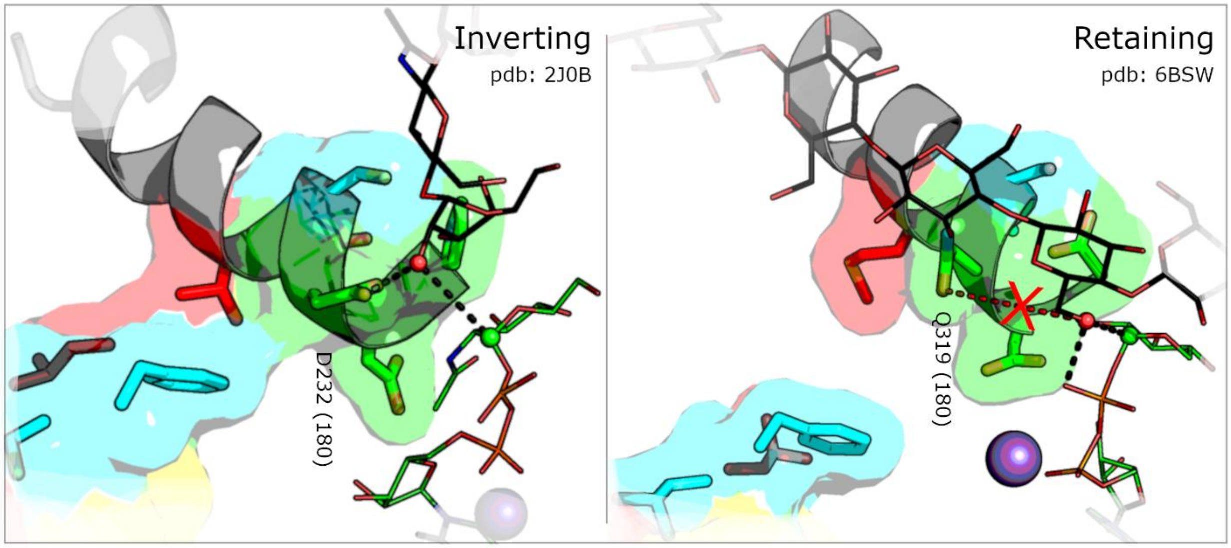

Figure S3: A comparison of representative inverting and retaining GT-A core packing in the same orientation, with substrates bound, as seen in Figure 4. The xED is highlighted in green, the C-lobe tether residues are highlighted in red, and in blue are residues in the logo adjacent to the C-lobe tether. Acceptor and donor substrates for the Inverting GT are analogs modeled from a structural alignment of a related enzyme, B3GNT2 (pdb: 6WMO). Dashes in black indicate a hydrogen bond distance. D232 deprotonates the acceptor nucleophile (red sphere), allowing for the anomeric carbon (green sphere) to attach via an S_n2 mechanism. The retaining GT acceptor has a red dash towards Q319 about five Angstroms in length, unlikely to deprotonate the nucleophile. Instead, it is deprotonated by the β-phosphate oxygen of the UDP, allowing the proximal anomeric carbon to attack via an S_ni mechanism.

Supplementary Figure 4

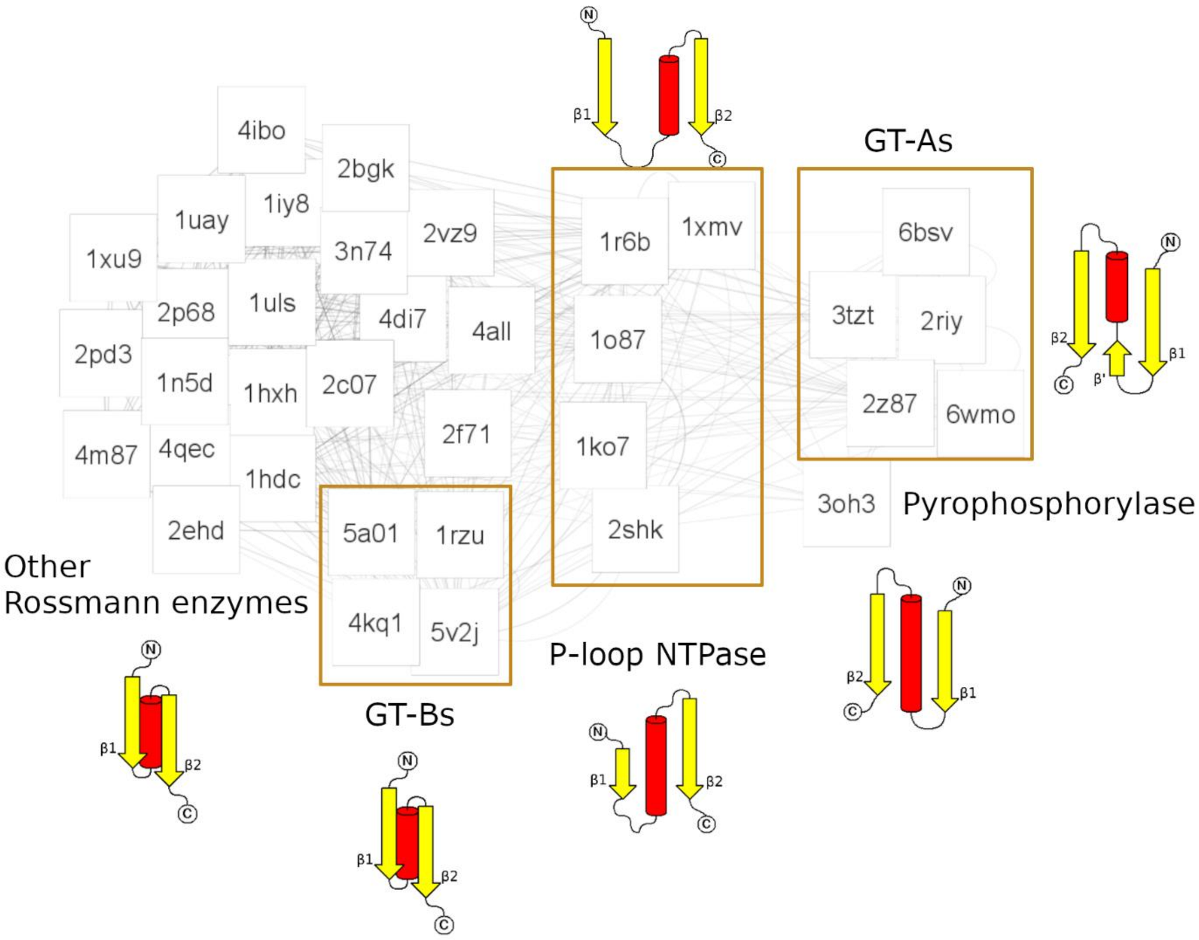

Figure S4: Cytoscape network of PBCs from different enzyme families clustered by RMSD after an all vs. all pairwise structural alignment. 2D cartoon topologies in each enzyme family are shown. Nodes are pdb IDs and edges are RMSD distances between nodes. The closest clustered family to GT-As are pyrophosphorylases. P-loop NTPases have different topologies and spatial arrangement in available crystal structures.

Supplementary Figure 5

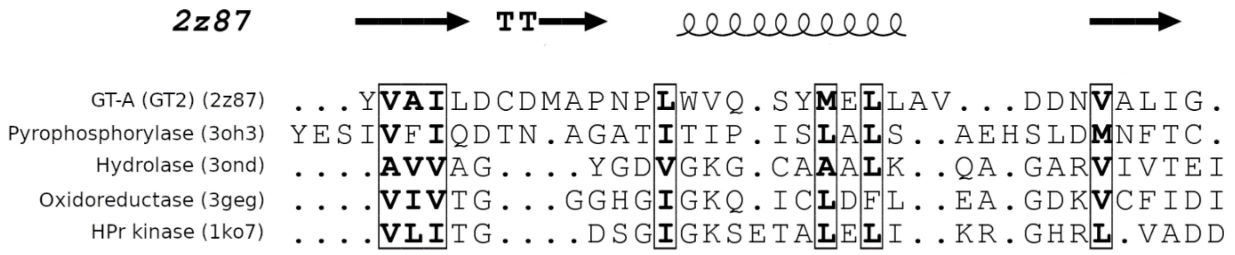

Figure S5: Structure based sequence alignment of PBC in representative families. Secondary structures are indicated above the alignment using GT2 as the template.

Supplementary Figure 6

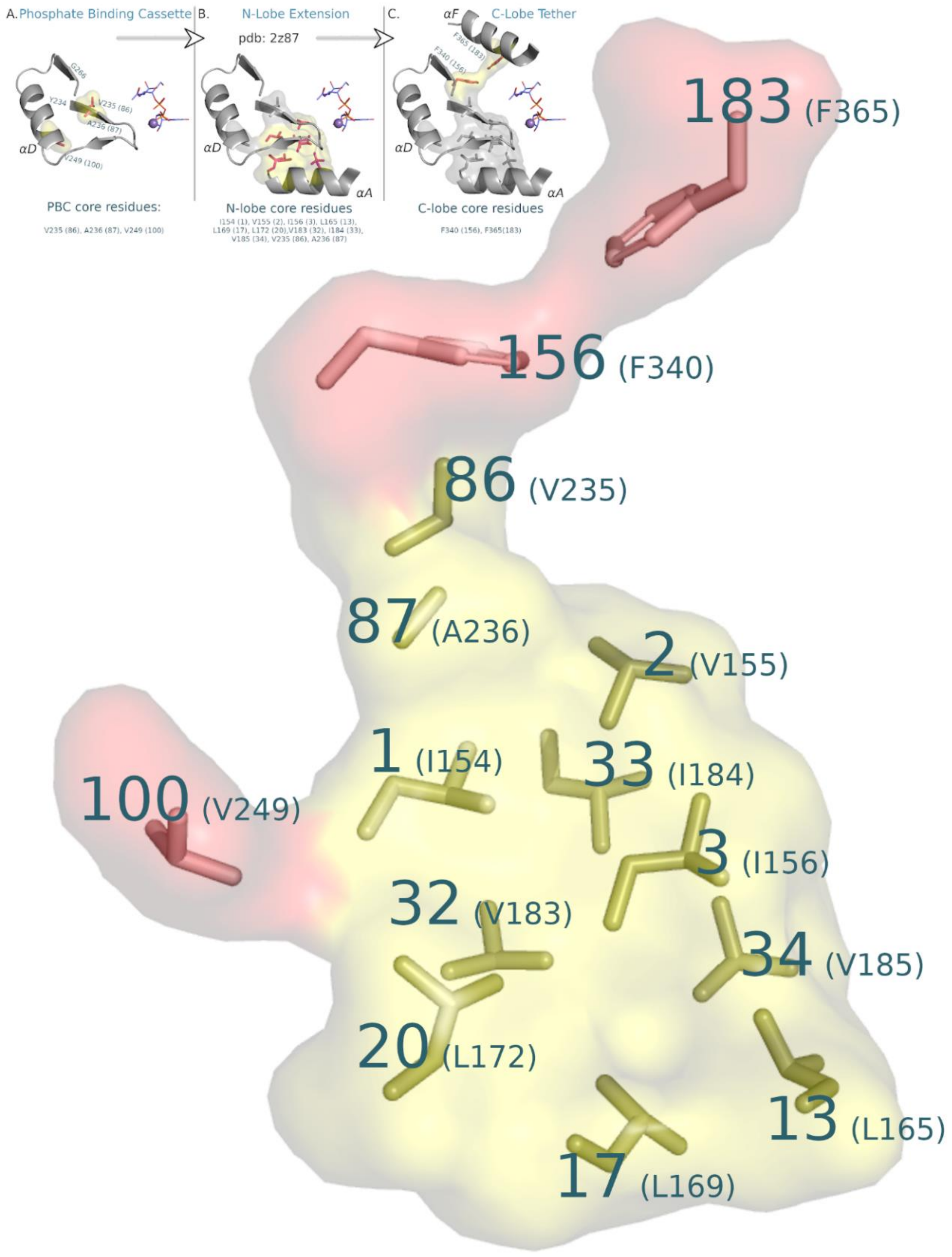

Figure S6: Hydrophobic core positions according to the previously published 231 aligned positions using pdb: 2z87. In yellow are residues conserved amongst Rossmann fold enzymes and in red are GT-A specific residues.

Supplementary Figure 7

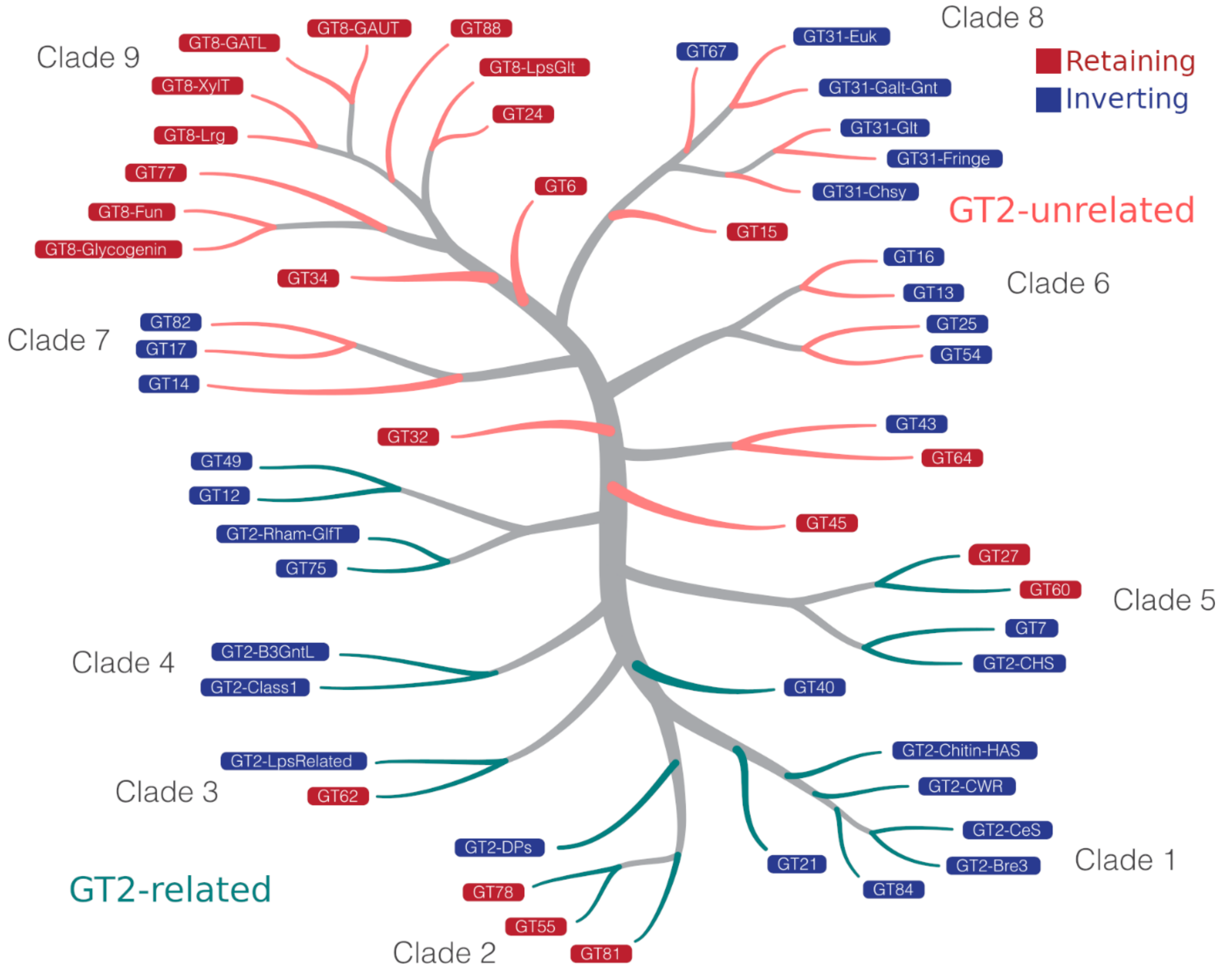

Figure S7: Phylogenetic tree showing the relationships connecting diverse GT-A fold enzymes. The tree is broadly classified into two halves, GT2-related and GT2-unrelated.

Supplementary Figure 8

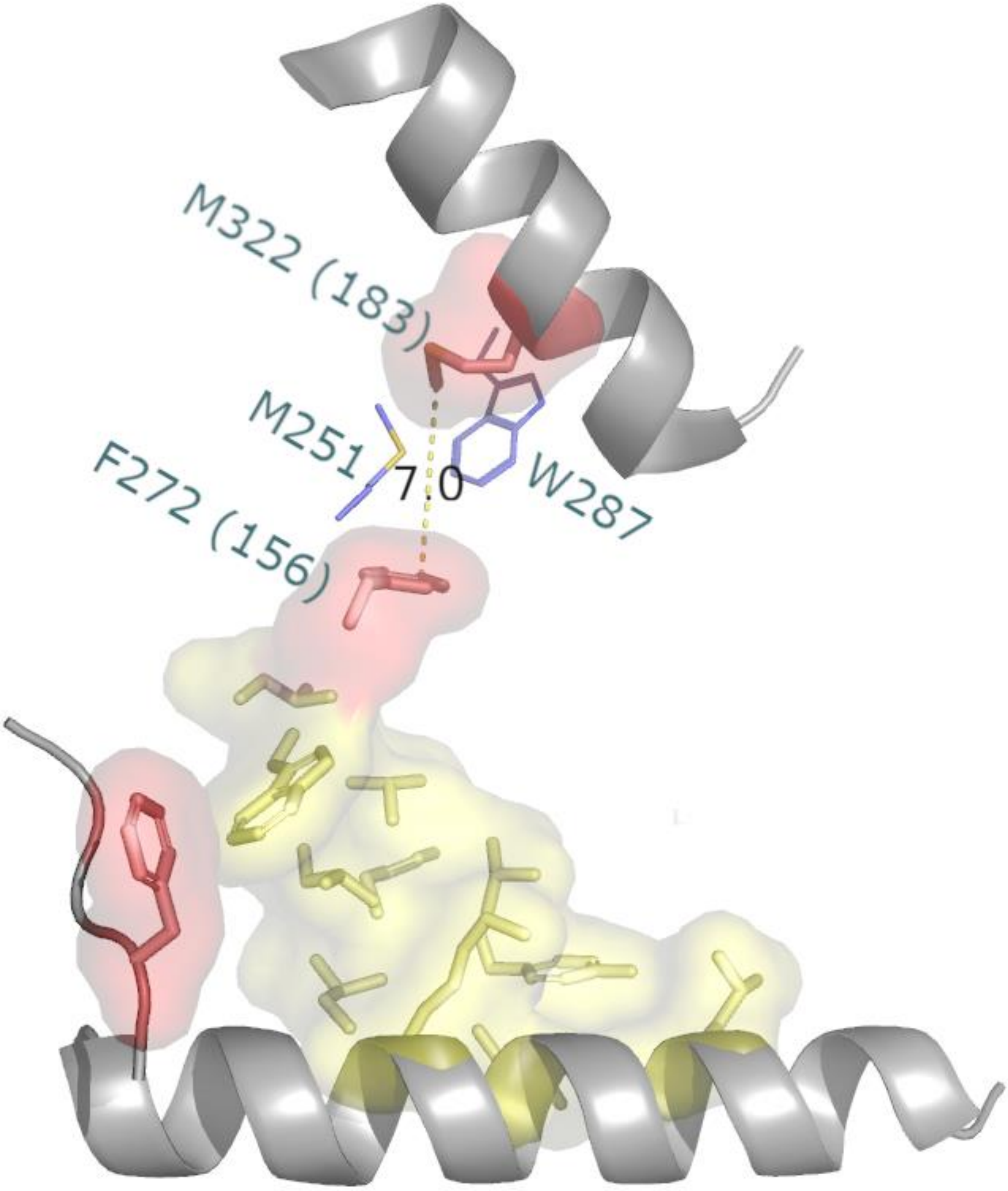

Figure S8: Extended hydrophobic network. Example of how a retaining GT-A (GT34, pdb: 6BSV) can rearrange packing to have a residue (or residues) from other parts of the enzyme aid in extending the C-lobe tether.

Supplementary Figure 9

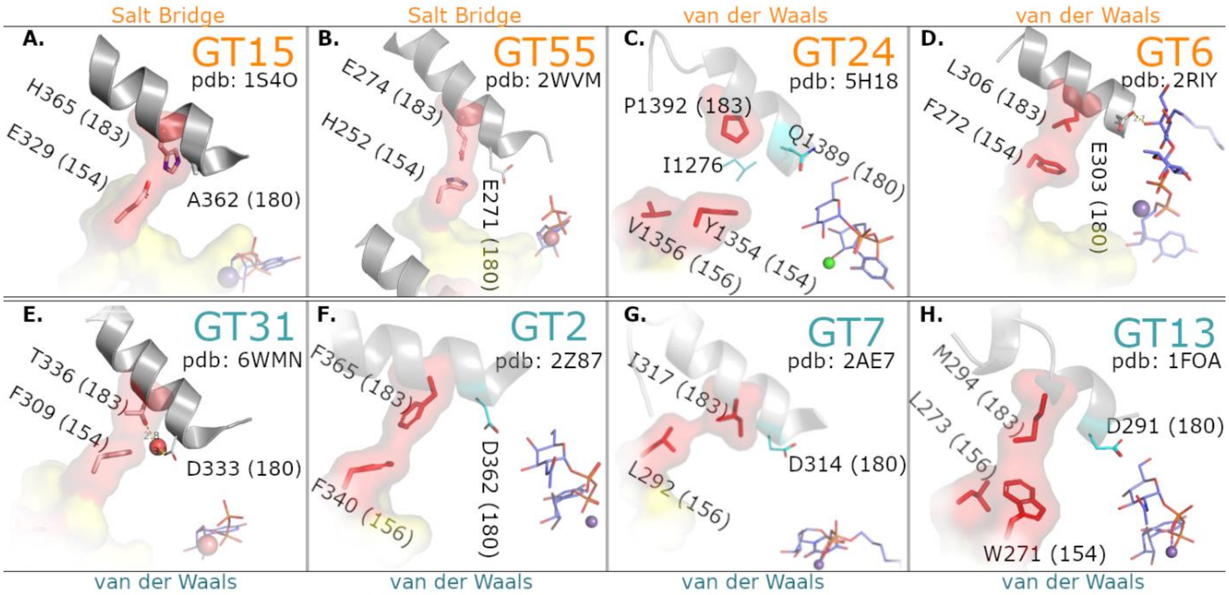

Figure S9: A-D) Representative retaining GT-As with unique family-specific variations in their tethers, with the type of interaction labeled above each GT-A. E-H) Representative inverting GT-As with unique family-specific variations in their tethers, with the type of interaction labeled below each GT-A.

Supplementary Figure 10

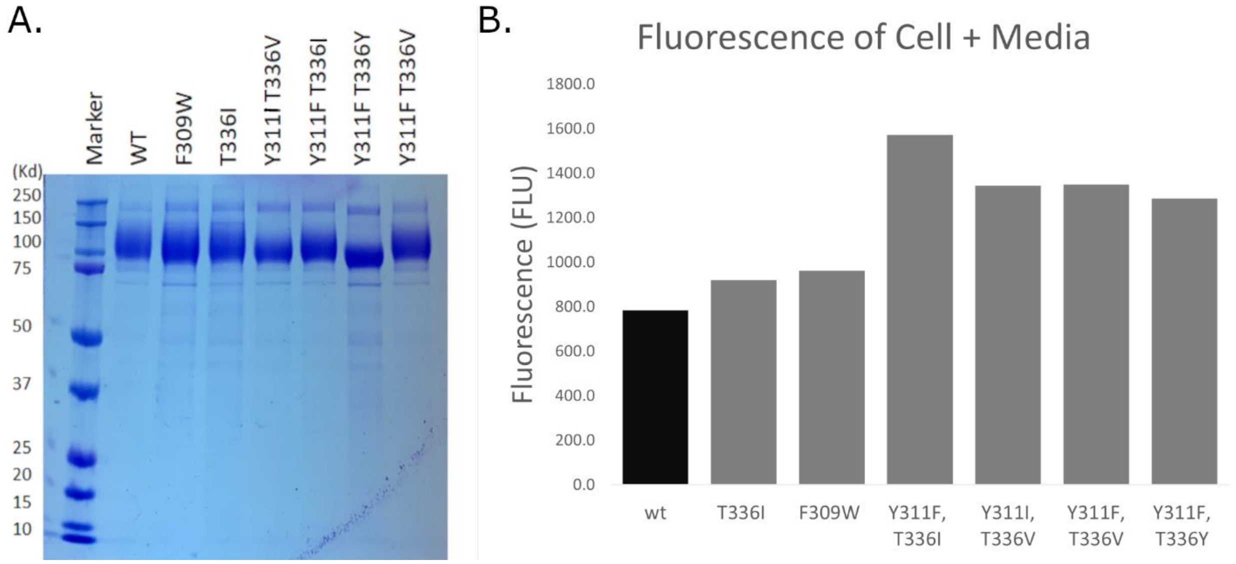

Figure S10: A) SDS-Page gel of the selected mutant B3GNT2-GFP fusion proteins, shown here all expressed in wild type HEK293 cells. The slight difference in molecular weight and band size is due to the differences in glycosylation patterns of the mutants. When B3GNT2 is expressed in wild type HEK293 cells, it contains 5 complex type N-glycans that can be extended with polylactosamine structures by the activity of the recombinant B3GNT2 itself (14). Wild type B3GNT2 and active mutants result in glycosylated proteins with an SDS-PAGE mobility considerably larger than the predicted 74kDa size of the polypeptide (>100 kDa). Mutants with lower activity (e.g., Y311F/T336Y) are less able to extend the polylactosamine structures and result in lower molecular weight species on the SDS-PAGE gel. B) Fluorescence data for the B3GNT2 variants expressed in HEK293 cells. The Fluorescence was measured from the crude media (cells and media) after transfection based on GFP-Fluorescence of the attached GFP-tag. Values are reported in Table S3.

Supplementary Figure 11

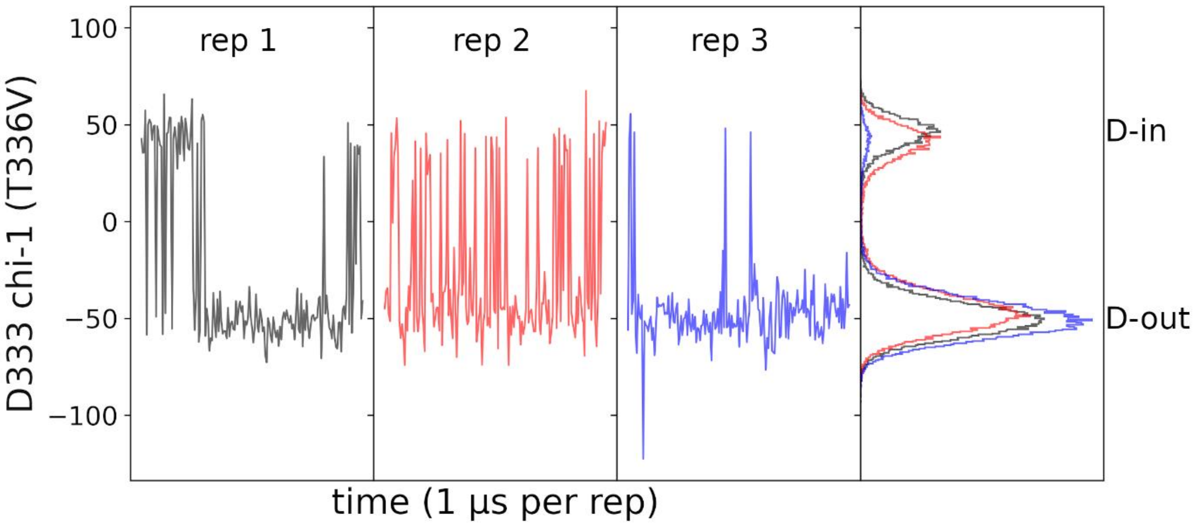

Figure S11: Additional three replicates of molecular dynamics simulations (one microsecond each) run on a T336V mutant, mimicking wt manic fringe. Distribution of D-in and D-out conformations observed in the MD are shown.

Supplementary Figure 12

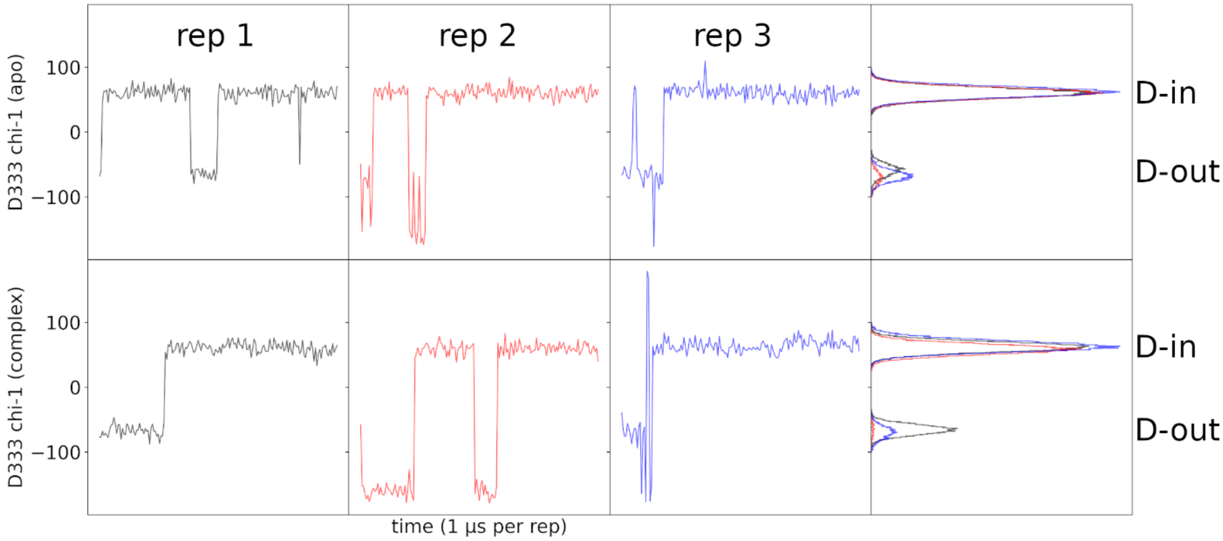

Figure S12: MD simulations with xED-aspartate protonated. Frequency of D-in and D-out conformations in the simulations are shown.

Supplementary Figure 13

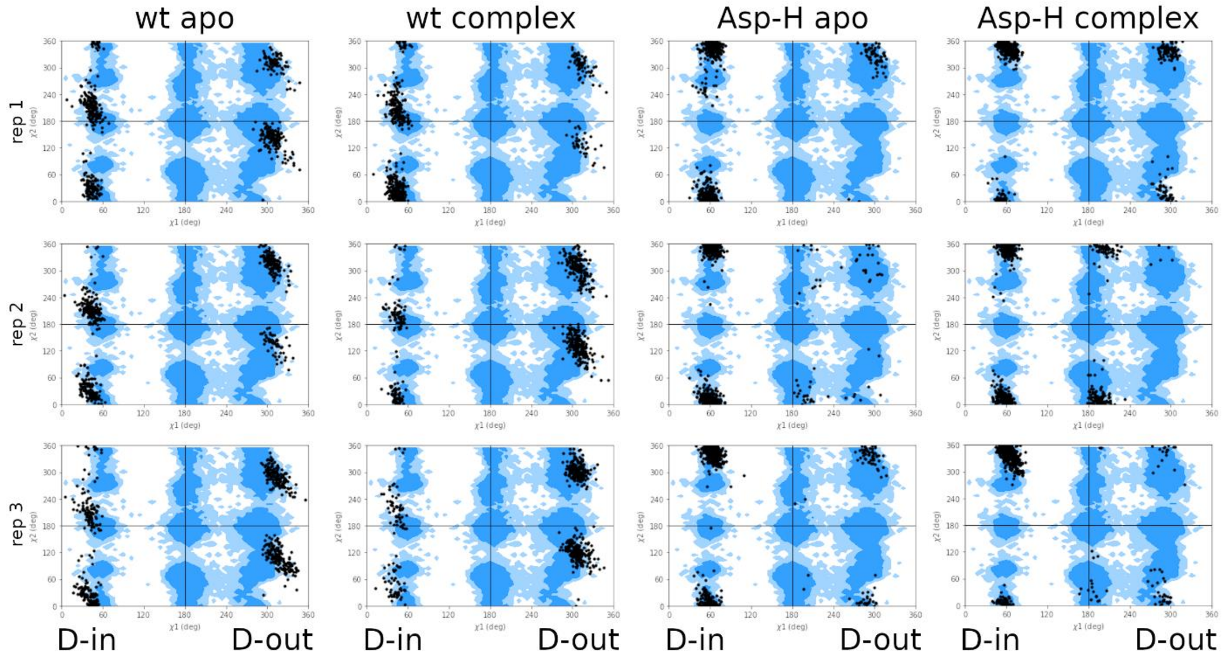

Figure S13: Frequency of chi-2 conformation of the xED-Asp in the protonated simulations. The D-in and D-out chi-1 conformations correspond to angles 60 and 300 (-60). Each point represents a D333 conformation sampled in one nanosecond of the microsecond MD replicates, plotted as a Janin plot (61), over a blue reference background that uses a previously published rotamer library (62).

Supplementary Figure 14

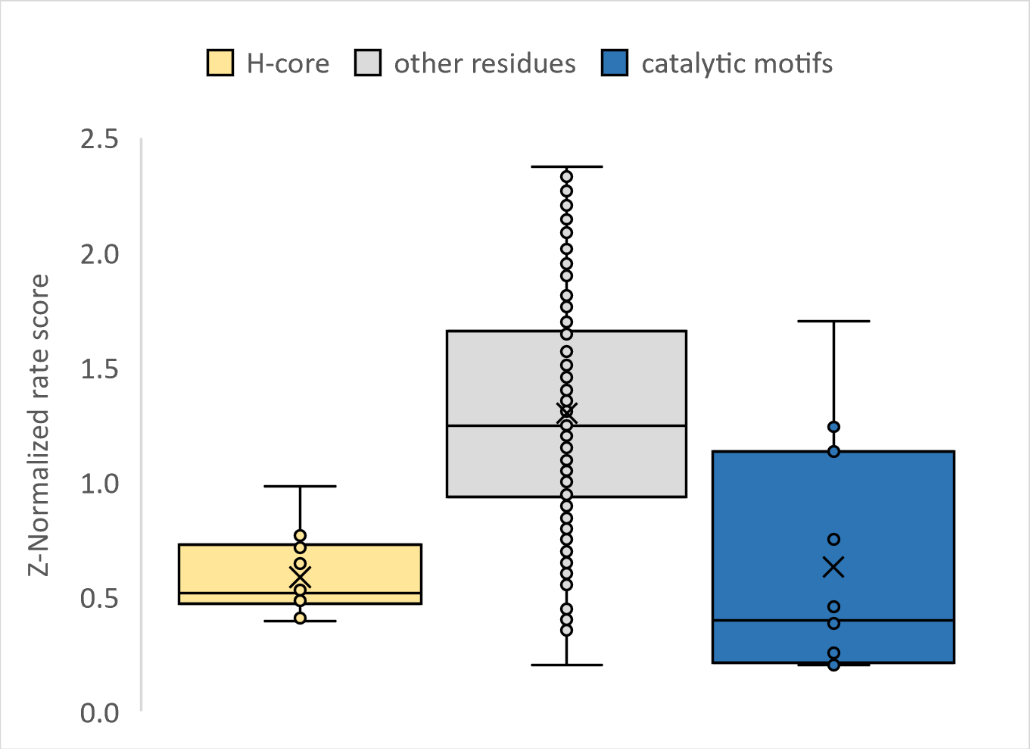

Figure S14: Boxplot of Z-scores of evolutionary rates of residues from Figure 7A organized as those belonging to the hydrophobic core, those that belong to key catalytic motifs (DxD, G-loop, xED, and C-His), and all other residues.

Supplementary Figure 15

Aligned Position	Cancer Counts
1	34
2	28
3	18
13	25
17	35
20	32
32	21
33	54
34	33
86	48
87	29
100	17
156	22
183	25

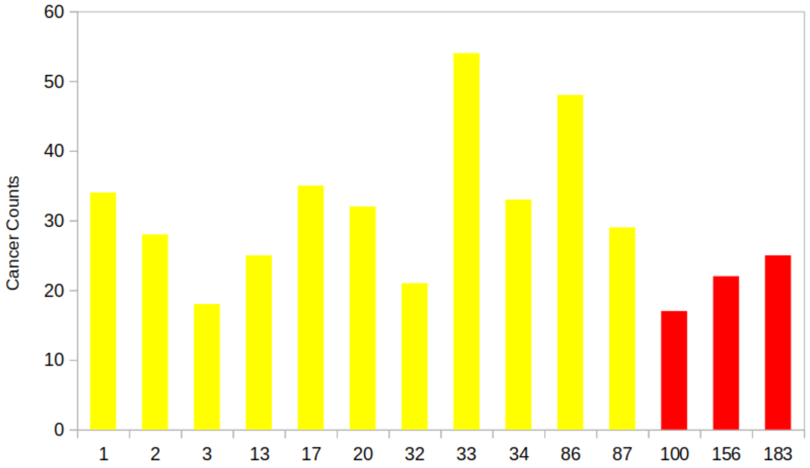

Figure S15: Aligned positions in the core along with respective counts of mutations found in cancers from TCGA for each region. The N-lobe hydrophobic core positions are in yellow, and the C-lobe tether positions are in red.

Supplementary Figure 16

	1	2	3	13	17	20	32	33	34	86	87	100	156	183
1														
2	1.3													
3	4.1	1.3												
13	8.9	8.6	5.9											
17	8.1	8.2	8.3	2.9										
20	8.9	8.1	11.2	7.5	3.2									
32	4.8	5.9	4.4	10.6	7.9	6.8								
33	5.4	5.4	5.1	10.1	8.1	9.4	1.3							
34	5.6	5.4	4.2	4.1	4.8	9.6	4.3	1.3						
86	4.3	5.3	8.9	15.3	13.5	13.0	9.6	6.7	9.8					
87	3.1	3.2	5.6	12.0	10.6	9.7	8.1	7.1	9.3	1.3				
100	9.9	11.7	14.0	14.9	11.3	6.8	7.9	11.7	16.0	9.6	5.4			
156	9.3	9.1	12.6	19.3	17.6	15.6	12.7	11.3	15.7	2.8	5.1	11.3		
183	13.2	14.0	12.8	20.0	20.7	19.6	19.4	17.3	18.2	10.6	10.7	15.8	7.8	

Figure S16: 2D representation of the median minimum distances (in Angstroms) between hydrophobic core positions in 118 representative pdbs. Because the distribution of distances was non-normal due to skew from outliers, median was used to better represent the data. A hydrophobic contact is generally a mid-range contact around 5-7 Angstroms (yellow). Residue interactions by van der waals or hydrogen bonding are shown in around the three angstrom range (green). Residues directly adjacent to each other are in the one angstrom range (blue).

Supplementary Figure 17

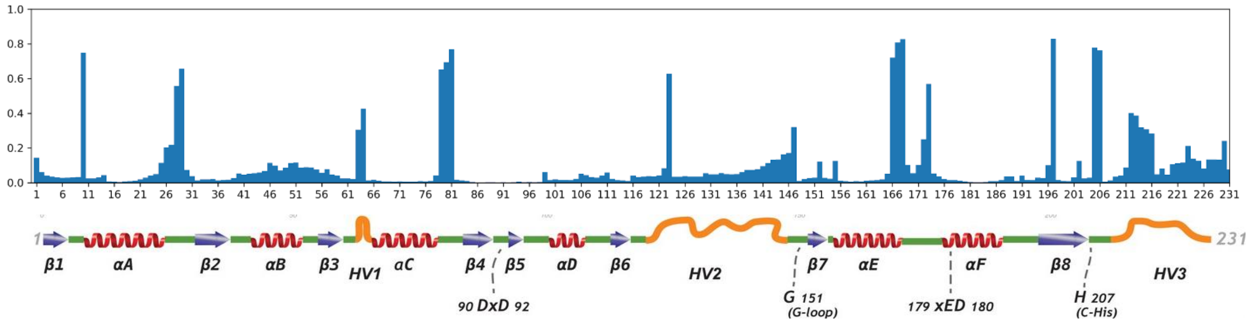

Figure S17: Deletions found across the aligned 231 positions in GT-As. The region with fewest deletions is the PBC (beta4 to beta6).

Supplementary Figure 18

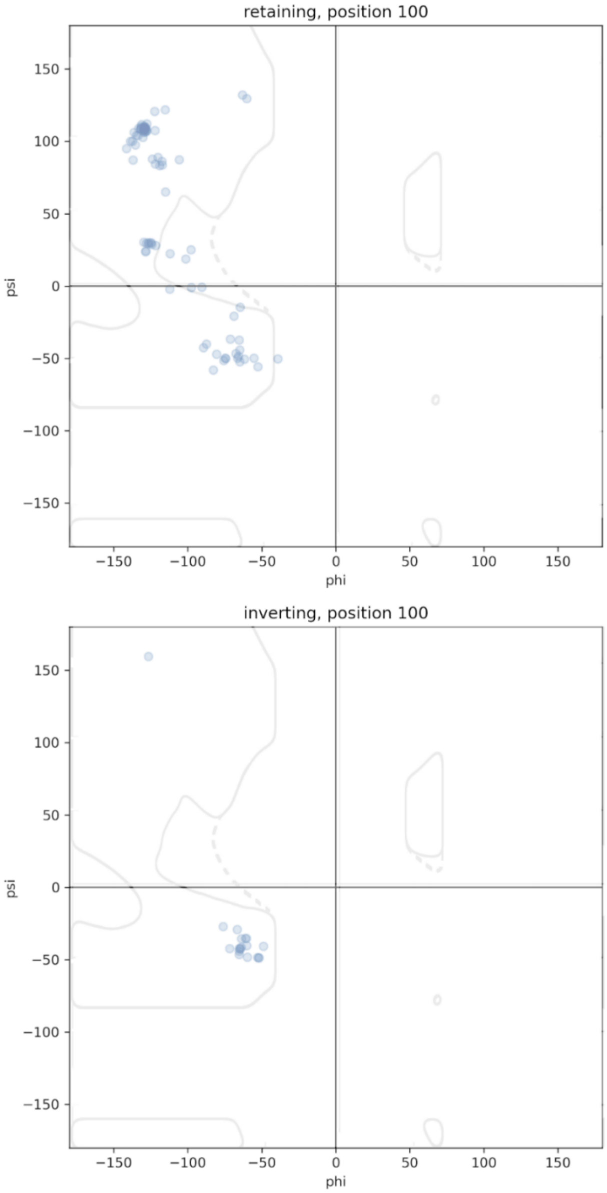

Figure S18: Ramachandran plot of angles for retaining and inverting GT-As at position 100 (a GT-A specific residue located on the PBC), based on a representative set of GT-As. The Ramachandran plot outline (63) was used to indicate ϕ,ψ-spaces of the residues at position 100. The retaining residues diverge from the inverting residues (with one outlier) with dense population at not only the 3-10/α helix regions, but the disfavored bridge and Beta sheet regions as well.

Supplementary Figure 19

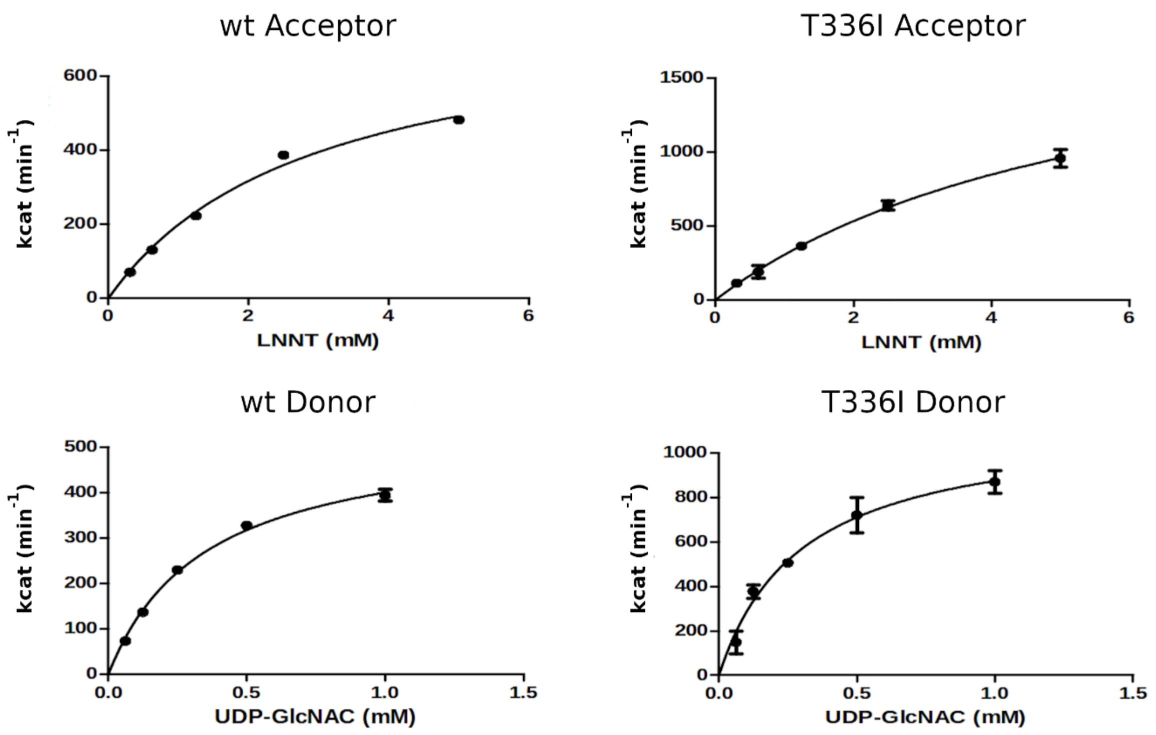

Figure S19: Kcat reaction kinetics plotted for wt and T336I mutants under acceptor and donor saturation.
